# Malaria vector species in Amazonian Peru co-occur in larval habitats but have distinct larval microbial communities

**DOI:** 10.1371/journal.pntd.0007412

**Published:** 2019-05-15

**Authors:** Catharine Prussing, Marlon P. Saavedra, Sara A. Bickersmith, Freddy Alava, Mitchel Guzmán, Edgar Manrique, Gabriel Carrasco-Escobar, Marta Moreno, Dionicia Gamboa, Joseph M. Vinetz, Jan E. Conn

**Affiliations:** 1 Department of Biomedical Sciences, School of Public Health, University at Albany–State University of New York, Albany, NY, United States of America; 2 Laboratorio ICEMR-Amazonia, Laboratorios de Investigación y Desarrollo, Facultad de Ciencias y Filosofia, Universidad Peruana Cayetano Heredia, Lima, Peru; 3 Wadsworth Center, New York State Department of Health, Albany, NY, United States of America; 4 Ministry of Health, Iquitos, Peru; 5 Facultad de Salud Pública, Universidad Peruana Cayetano Heredia, Lima, Peru; 6 Division of Infectious Diseases, Department of Medicine, University of California San Diego, La Jolla, CA, United States of America; 7 Departamento de Ciencias Celulares y Moleculares, Facultad de Ciencias y Filosofía, Universidad Peruana Cayetano Heredia, Lima, Peru; 8 Instituto de Medicina Tropical “Alexander von Humboldt”, Universidad Peruana Cayetano Heredia, Lima, Peru; Vienna, AUSTRIA

## Abstract

In Amazonian Peru, the primary malaria vector, *Nyssorhynchus darlingi* (formerly *Anopheles darlingi*), is difficult to target using standard vector control methods because it mainly feeds and rests outdoors. Larval source management could be a useful supplementary intervention, but to determine its feasibility, more detailed studies on the larval ecology of *Ny*. *darlingi* are essential. We conducted a multi-level study of the larval ecology of Anophelinae mosquitoes in the peri-Iquitos region of Amazonian Peru, examining the environmental characteristics of the larval habitats of four species, comparing the larval microbiota among species and habitats, and placing *Ny*. *darlingi* larval habitats in the context of spatial heterogeneity in human malaria transmission. We collected *Ny*. *darlingi*, *Nyssorhynchus rangeli* (formerly *Anopheles rangeli*), *Nyssorhynchus triannulatus* s.l. (formerly *Anopheles triannulatus* s.l.), and *Nyssorhynchus* sp. nr. *konderi* (formerly *Anopheles* sp. nr. *konderi*) from natural and artificial water bodies throughout the rainy and dry seasons. We found that, consistent with previous studies in this region and in Brazil, the presence of *Ny*. *darlingi* was significantly associated with water bodies in landscapes with more recent deforestation and lower light intensity. *Nyssorhynchus darlingi* presence was also significantly associated with a lower vegetation index, other Anophelinae species, and emergent vegetation. Though they were collected in the same water bodies, the microbial communities of *Ny*. *darlingi* larvae were distinct from those of *Ny*. *rangeli* and *Ny*. *triannulatus* s.l., providing evidence either for a species-specific larval microbiome or for segregation of these species in distinct microhabitats within each water body. We demonstrated that houses with more reported malaria cases were located closer to *Ny*. *darlingi* larval habitats; thus, targeted control of these sites could help ameliorate malaria risk. The co-occurrence of *Ny*. *darlingi* larvae in water bodies with other putative malaria vectors increases the potential impact of larval source management in this region.

## Author summary

The standard methods used to combat mosquitoes that transmit malaria, long-lasting insecticide treated nets and indoor residual spraying, target mosquitoes that bite people indoors and rest indoors after biting. In Amazonian Peru, the major malaria vector, *Nyssorhynchus darlingi* (formerly *Anopheles darlingi*), is known to bite and rest mostly outdoors and to feed on animals as well as humans. Therefore, additional methods are needed to control this species, such as targeting immature mosquitoes in water bodies using chemical or biological larvicides or environmental modification. To determine whether this is feasible, more ecological information about *Ny*. *darlingi* larval habitats in this region is needed. In this study, we found that *Ny*. *darlingi* were more likely to be collected from water bodies in more deforested areas, and in the presence of other species of mosquitoes that transmit malaria. We characterized the bacteria detected in three species of mosquito larvae, and found that the species of mosquito, and not the water body, determined which bacteria were identified. As we found that houses with more malaria cases were located closer to *Ny*. *darlingi* larval habitats, management of larval habitats may be an effective method to reduce the risk of malaria in this region.

## Introduction

Despite substantial progress in reducing the global burden of malaria over the last two decades, no progress was made in decreasing the total number of malaria cases worldwide between 2015 and 2017 [1]. This emphasizes the need not only for continued commitment to the two most effective malaria vector control methods, long-lasting insecticidal nets (LLINs) and indoor residual spraying (IRS), but also for the use of alternative vector control methods [2, 3]. LLINs and IRS target mainly mosquitoes that feed and rest indoors (endophagic and endophilic, respectively). To combat residual malaria transmission that occurs despite universal coverage with LLINs and/or IRS, vector interventions need to incorporate tools targeting mosquitoes that feed and rest outdoors (exophagic and exophilic, respectively) [4]. In this study we focus on the main Amazonian malaria vector, *Nyssorhynchus darlingi* Root (formerly *Anopheles darlingi* [5]), which displays a high degree of plasticity in its biting behavior [6–9], making it difficult to target with LLINs and IRS.

Larval source management, used frequently in high-income countries for vector control, targets immature mosquitoes, and therefore its efficacy does not depend on the biting behavior of adult mosquitoes [10, 11]. Larval source management is currently recommended as a supplementary malaria vector control method by the WHO only in areas where larval habitats are “few, fixed, and findable” [12]. To evaluate whether larval source management will be effective in a malaria endemic region in the Amazon, and to plan targeted larval source management strategies to maximize the use of limited resources, it is necessary to have at least a basic understanding of the larval ecology of local and regional malaria vectors, a topic that is understudied globally [10, 12–14].

In Peru, over 90% of malaria cases occur in the Loreto Department, where malaria is endemic and seasonal, peaking during the rainy season [15, 16]. *Plasmodium vivax* causes the majority of regional malaria cases, but the proportion of cases caused by *Plasmodium falciparum* has been increasing since 2011 [16–18]. The primary malaria vector in Loreto is *Ny*. *darlingi*, reintroduced into the region in the 1990s following elimination in the 1960s [19]. A comprehensive survey of *Ny*. *darlingi* larval habitats in the peri-Iquitos region of Loreto in 2000–2001 focused only on water bodies along the Iquitos-Nauta Highway [20]. Our group has found previously that adult *Ny*. *darlingi* in riverine and highway environments in peri-Iquitos differ in their biting behavior and exhibit genetic differentiation [21]. An unanswered question is whether there are unique characteristics of riverine or highway larval habitats that contribute to such differentiation. Vittor et al. found that *Ny*. *darlingi* larvae in peri-Iquitos were associated with large water bodies, filamentous algae, the presence of human populations, and lower forest cover [20]. Elsewhere in its distribution, *Ny*. *darlingi* larvae have similarly been associated with human-modified habitats, including ponds constructed for fish farming and water bodies near the fringes of forested areas [22–25]. In peri-Iquitos, fish farming is common, and increased density of fish ponds has been associated with an increased risk of malaria [26], though a clear link between *Ny*. *darlingi* larvae and fish ponds has not been demonstrated [20]. To investigate these associations, and to determine how they relate to the risk of malaria, more current research on the relationship between *Ny*. *darlingi* and human-modified larval habitats in the peri-Iquitos region, including deforested sites and fish ponds, is necessary.

The composition of the microbiota of Neotropical malaria vectors is another critical knowledge gap. Mosquitoes rely on midgut microbiota for development [27], and the composition of the *Anopheles gambiae* microbiome affects its vector competence for *P*. *falciparum* [28, 29]. In the Neotropics, a few studies have focused on the *Nyssorhynchus albimanus* Wiedemann (formerly *Anopheles albimanus* [5]) microbiota [30–32], and until recently, only limited studies of *Ny*. *darlingi* using Sanger sequencing or culture-based methods on small numbers of mosquitoes had been published [33–35]. A recent study using next-generation 16S sequencing characterized the microbial composition of a large sample of *Ny*. *darlingi* and *Nyssorhynchus nuneztovari* s.s. Gabaldón (formerly *Anopheles nuneztovari* s.s. [5]) larvae and adults collected in coastal Colombia [36]. This study found that mosquito developmental stage and geographic location, but not mosquito species, influenced the composition of the gut microbiota. In mosquitoes from Africa and the United States, bacterial communities have been shown to differ across larval habitats or localities [36–40], between seasons [40, 41] and between species [39, 42, 43]. Where mosquito species share larval habitats, the extent to which the larval environment vs. mosquito species influences microbiome composition has varied across studies and may be different across systems. No studies addressing this question have been conducted in malaria vectors in the Peruvian Amazon.

Several other species of *Nyssorhynchus* that may be acting as secondary malaria vectors are also found in the peri-Iquitos region. These include *Nyssorhynchus triannulatus* s.l. Neiva & Pinto (formerly *Anopheles triannulatus* s.l. [5]), *Nyssorhynchus rangeli* Gabaldón, Cova-García & López (formerly *Anopheles rangeli* [5]), and members of the *Nyssorhynchus* Oswaldoi-Konderi complex (formerly the *Anopheles* Oswaldoi-Konderi complex [5]). *Nyssorhynchus triannulatus* s.l. is a species complex distributed throughout Latin America and the Caribbean that has been incriminated as a human malaria vector [44, 45]. *Nyssorhynchus rangeli* is distributed throughout the Amazon Basin and is a local vector of *Plasmodium* in southern Colombia [46] and Junín Department, Peru [47]. The Oswaldoi-Konderi complex consists of five species broadly distributed throughout South America, several of which have been implicated as malaria vectors [48]: *Nyssorhynchus oswaldoi* s.s. Peryassú (formerly *Anopheles oswaldoi* s.s. [5]), *Nyssorhynchus oswaldoi* A Ruiz-Lopez (formerly *Anopheles oswaldoi* A [5]), *Nyssorhynchus oswaldoi* B Ruiz (formerly *Anopheles oswaldoi* B [5]), *Nyssorhynchus konderi* Galvão & Damasceno (formerly *Anopheles konderi* [5]), and *Nyssorhynchus* sp. nr. *konderi* Ruiz-Lopez (formerly *Anopheles* sp. nr. *konderi* [5]).

For the current study, we collected Anophelinae mosquito larvae longitudinally from water bodies in eight villages on four rivers and one highway in the peri-Iquitos region to investigate the environmental drivers of differences in the composition of larval communities within water bodies and of bacterial communities within larvae in the context of malaria risk. Our aims were: 1) to characterize the larval habitats of malaria vectors in peri-Iquitos and test the hypothesis that *Ny*. *darlingi* larvae are associated with human-modified habitats; 2) to determine whether the spatial distribution of malaria cases is associated with the spatial distribution of *Ny*. *darlingi* larval habitats; and 3) to describe the larval microbiota of three malaria vectors and test the hypothesis that its composition is habitat-specific.

## Methods

### Ethics statement

Authorization for the fieldwork included in this study was given by the Dirección de Gestión Forestal y de Fauna Silvestre and the Dirección General Forestal y de Fauna Silvestre of the Ministerio de Agricultura de la República del Perú, permit N. 0424-2012-AG-DGFFS-DGEFFS.

### Study villages

**Fig 1:**
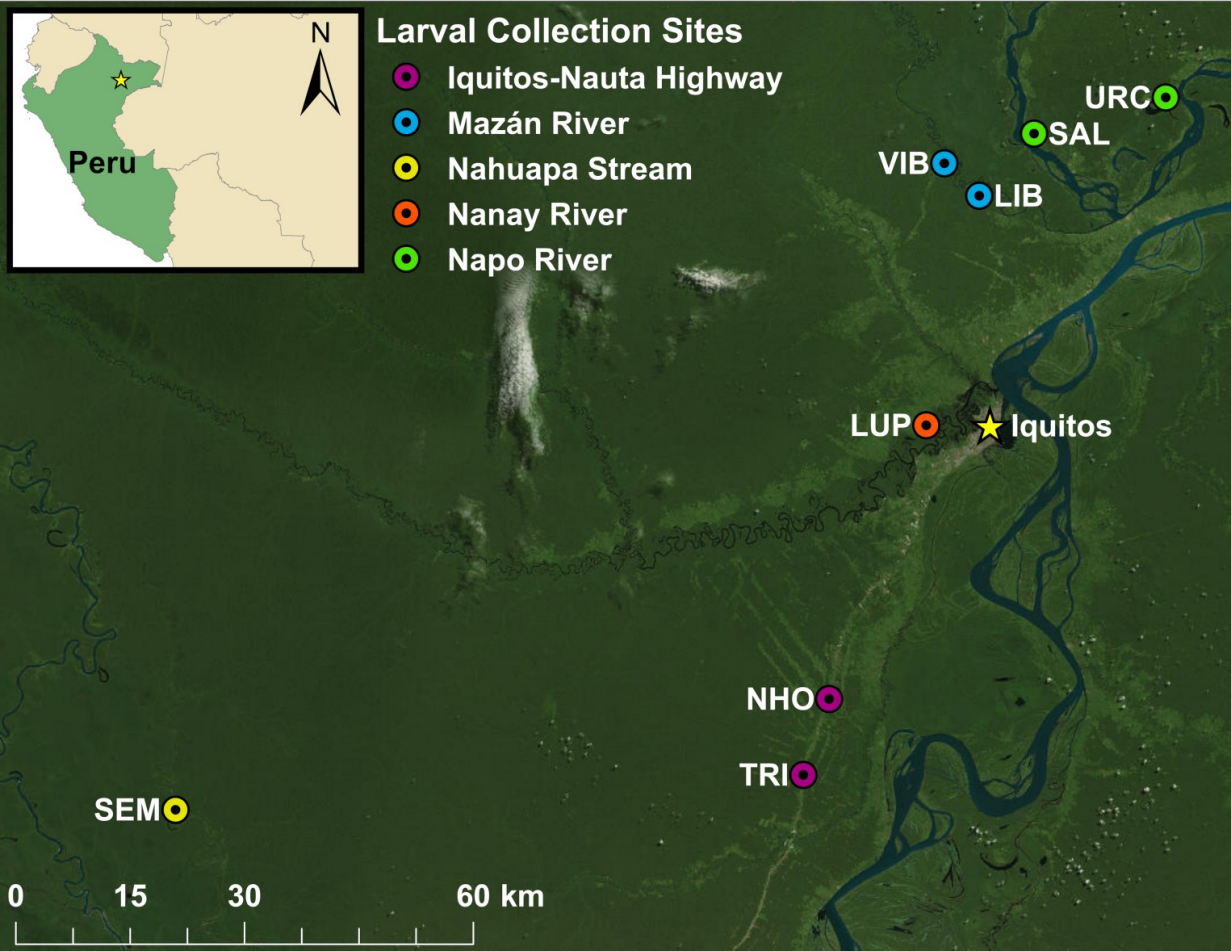
Map of villages in which larval collections were conducted in the peri-Iquitos region. Colors indicate river or highway on which each village is located. LIB: Libertad, LUP: San José de Lupuna, NHO: Nuevo Horizonte, SAL: Salvador, SEM: Santa Emilia, TRI: El Triunfo, URC: Urco Miraño, VIB: Visto Bueno. Basemap: ESRI World Imagery.

Anophelinae larvae were collected from eight villages in the peri-Iquitos region of Loreto, Peru (Fig 1). Four villages to the south and west of Iquitos (San José de Lupuna (LUP) on the Nanay River, Santa Emilia (SEM) on the Nahuapa Stream, and Nuevo Horizonte (NHO) and El Triunfo (TRI) on the Iquitos-Nauta Highway) have been described previously [21]. The four villages north of Iquitos are located in the Mazán District, that consists of small communities largely supported by agriculture, fishing, and timber extraction, and has a high overall incidence of malaria [49, 50]. In 2017, 1000 cases of *P*. *vivax* and 349 cases of *P*. *falciparum* were reported in the Mazán District (Annual Parasite Index (API) of 96.1 per 1000 inhabitants [51]). The four villages in the current study were selected because they each had an API greater than 10 cases per 1000 inhabitants, and to represent two ecologically distinct river systems [52]. Salvador (SAL) and Urco Miraño (URC) are on the Napo River, a large white water river that originates in Ecuador and flows into the Amazon River; and Libertad (LIB) and Visto Bueno (VIB) are on the Mazán River, a black water river that is a tributary of the Napo River.

### Larval collections

In each village, collections were conducted 5–6 times over the study period (Table 1). Satellite images were used to identify water bodies within a 1km radius of each village (to correspond to the approximate flight range of *Ny*. *darlingi* [7]), and additional water bodies in this radius were identified by ground-truthing. Mosquito larvae dippers (350mL capacity) were used to sample each water body once per collection. Sampling points were selected 10m apart along the perimeter of each water body, with at most 20 sampling points per water body. Ten dips were taken at each sampling point and examined for the presence of Anophelinae larvae.

**Table 1:** Dates of 2016–17 larval collections in eight peri-Iquitos region villages.

Collection	South/West villages (LUP, SEM, NHO, TRI)	Mazán District villages (SAL, URC, LIB, VIB)
**1**	January/February 2016*	March 2016*
**2**	April 2016*	June 2016*
**3**	July/August 2016	September 2016
**4**	September/October 2016	November 2016
**5**	November/December 2016	February/March 2017*
**6**	January-March 2017*	

*Denotes rainy season collections; all others dry season

Characteristics of each water body were recorded at the time of each collection, including type of water body (fish ponds were recorded as active or abandoned); depth; cloud cover; shade level; presence of vegetation, fish, and amphibians; water movement; type of bed material; and light intensity (Foot Candle/Lux meter, Extech, Nashua, NH, USA). Additionally, the alkalinity, hardness, and nitrate and nitrite levels (Eco-Check 5-in-1 Test Strips, Industrial Test Systems, Inc., Rock Hill, SC, USA); and pH, temperature, conductivity and salinity (ExStik pH/Conductivity Meter, Extech, Nashua, NH, USA) of each water body was recorded.

### Species identification

Larvae collected in LUP, NHO, TRI, and SEM were reared to adults for species identification using morphological keys [53–55]. If the reared larvae died before adulthood, were not able to be identified, or were identified as a species other than *Ny*. *darlingi*, they were preserved in 100% ethanol (for larvae) or on silica gel (for adults) for molecular identification. All larvae collected in LIB, SAL, URC, and VIB were killed and preserved in 100% ethanol immediately after collection.

For molecular identification, total genomic DNA was extracted from whole larvae and reared adults using the DNeasy Blood & Tissue kit (Qiagen, Hilden, Germany). Mosquitoes were identified by PCR-RFLP of the ribosomal internal transcribed spacer 2 (ITS2) region [56] or, if the ITS2 region did not amplify, did not digest, or did not have an identifiable RFLP pattern, by cytochrome c oxidase subunit I (*COI*) barcode sequencing [57, 58]. Sequences of primers used for the ITS2 PCR-RFLP and for *COI* sequencing are included in Table I in S1 File. The *COI* barcode region was sequenced in one direction using the forward primer at the Wadsworth Center Applied Genomic Technologies Core (New York State Department of Health). All unique *COI* sequences were deposited in GenBank (accession numbers MK172893 to MK173015; *Nyssorhynchus dunhami* Causey (formerly *Anopheles dunhami* [5]) *COI* sequences were previously deposited in GenBank under accession numbers MH723612 to MH723658). Identifications were done by querying the sequences against the BOLD Identification System [59] or GenBank (https://www.ncbi.nlm.nih.gov/genbank/). Only larvae identified as species in the genera *Anopheles*, *Kerteszia*, *Lophopodomyia*, *Nyssorhynchus*, or *Stethomyia* (all formerly subgenera in the genus *Anopheles* [5]) were included in the analysis (S1 Dataset).

*COI* sequencing was done for all samples identified by ITS2-PCR-RFLP as members of the Oswaldoi-Konderi complex. Sequences were edited and checked for stop codons and pseudogenes using Geneious v9.1.4 [60]. Oswaldoi-Konderi complex (*Ny oswaldoi* s.s., *Ny*. *oswaldoi* A, *Ny*. *oswaldoi* B, *Ny*. *konderi*, and *Ny*. sp. nr. *konderi*) *COI* sequences from Ruiz et al. [61] and Saraiva et al. [48] were retrieved from GenBank and aligned to Oswaldoi-Konderi complex larval sequences from this study, using default settings of MUSCLE [62] in MEGA 7v7.0.26 [63]. Sequences were trimmed to 578 bp and a haplotype file created with DAMBE6 [64] (S2 Dataset). These data were used to construct a median-joining haplotype network in POPART v1.7 [65], with epsilon set to 0. Based on this haplotype network (Fig I in S1 File), one individual from this study was identified as *Ny*. *konderi*, while the remaining individuals were identified as *Ny*. sp. nr. *konderi*.

### Remote sensing analysis

Enhanced Vegetation Index (EVI), Normalized Difference Vegetation Index (NDVI) and Normalized Difference Water Index (NDWI) were calculated based on Landsat8 collections in Google Earth Engine repositories (Landsat 8 8-day EVI, NDVI and NDWI composites). These Landsat 8 composites are constructed from Level L1T orthorectified scenes, using the computed top-of-atmosphere (TOA) reflectance [66]. The collections have a 30m spatial resolution, and 8-day temporal resolution; between 3 to 4 images per month. EVI was generated from the Near Infra-Red (NIR), Red and Blue bands of each scene [67]. NDVI was generated from the NIR and Red bands of each scene as (NIR—Red) / (NIR + Red). NDWI is sensitive to changes in liquid water content of vegetation canopies. It is derived from the NIR band and a second Infra-Red (IR) band, ≈1.24μm when available or the nearest available IR band [68]. All normalized indices range from -1.0 to 1.0. Mean EVI, NDVI and NDWI were calculated within four buffers of 50, 100, 250 and 500m radius constructed around each water body at each collection. A two-month window was used when images were not available due to dense cloud coverage.

The distance from each water body to the closest forest patch was calculated in QGIS 3.0. Forest fringes were delimited by manual digitalization based on visual inspection of Google Earth imagery; any tree patch containing greater than 10m^2^ canopy cover was considered a forest patch. The dates of available imagery for each village are shown in Table II in S1 File. The resulting polygons that delineate the forest fringes were imported into QGIS along with the coordinates of the water bodies. A proximity raster was generated based on the rasterization of the forested area polygons. Briefly, in each cell, the distance to a target point was calculated using the proximity algorithm. As result, a raster with the minimum distance to the forest areas was generated for the entire study area and the values for each water body in meters were extracted.

The Hansen Global Forest Change dataset, the result of a time-series analysis of Landsat images that characterizes global forest extent and change [69], was used to compute forest cover and forest loss in Google Earth Engine. Yearly forest cover and forest loss area were calculated around each water body at different buffer sizes (50, 100, 250 and 500m) from 2010 to 2016. Percent forest cover in 2016 (the forest cover area divided by the total area of the buffer) and percent forest loss between 2010 and 2016 (the difference between the forest cover area in 2010 and 2016 divided by the forest area in 2010) at each buffer size was calculated in R v.3.5.3.

### Larval habitat analysis

To determine whether *Nyssorhynchus* species larvae were co-occurring more often than would be expected by chance, affinity indices between pairs of the five most common *Nyssorhynchus* species were calculated using the formula described by Fager and McGowan [70]: $\frac{J}{\sqrt{N_{A}*N_{B}}}\times \;\frac{1}{2\;\sqrt{N_{B}}}$, where *J* is the number of collection points (water bodies sampled at a collection) at which the species are both present, *N*_*A*_ is the total number of collection points at which species A is present, and *N*_*B*_ is the total number of collection points at which species B is present, chosen so that N_A_ ≤ N_B_. An affinity index ≥0.5 indicates affinity between the species.

The characteristics of *Ny*. *dunhami* larval habitats from this study have been described [71]. The water body characteristics associated with the presence of the four most abundant species excluding *Ny*. *dunhami* were evaluated separately for each species at the level of the collection point (S3 Dataset). Unless otherwise indicated, all data processing and analysis described in this section was done in R v. 3.5.2 [72]. Collection points at which a water body was dry at the time of collection were excluded from the analysis. If a water body was dry at one or more collections, it was considered temporal, otherwise, it was considered permanent. The distance from each water body to the nearest non-dry water body, and to the nearest water body positive for *Ny*. *darlingi* at least once during this study, were calculated using the R package ‘sp’ [73].

Censuses that determined the number of people living in each georeferenced house were completed in May 2015 in SEM, November 2015 in LUP, and November/December 2016 in all other villages. Census data were used to calculate the distance between each water body and the nearest inhabited house, and the number of people living within 50, 100, 250, and 500m radius buffers from each water body (also used to calculate presence/absence variables for whether any people lived within each buffer) using the R packages ‘sp’ [73] and ‘rgeos’ [74].

Independent variables evaluated for association with the presence of each *Nyssorhynchus* species are listed in Table 2. Nitrate and nitrite levels were nearly always zero (99.3% and 99.8% of collection points, respectively), so they were not included. Alkalinity and hardness values were recoded as zero/non-zero to account for rare levels.

**Table 2:** Larval habitat characteristics evaluated for associations with presence of *Nyssorhynchus* species.

Category	Variable	Variable type
General characteristics	Village	Discrete
	Quarter of the year (January-March 2016 etc.)	Discrete
	Water body type	Discrete
Terrestrial characteristics	Trees	Binary
	Bushes	Binary
	Grass	Binary
	Shade level (total, partial, none)	Discrete
	Cloud cover	Binary
	Light intensity	Continuous
	Distance to nearest inhabited house	Continuous
	Number of people living within buffer (50/100/250/500m)	Continuous
	Any people living within buffer (50/100/250/500m)	Binary
	EVI, NDVI, NDWI (50/100/250/500m buffers)	Continuous
	Distance to nearest non-dry water body	Continuous
	Distance to nearest *Ny*. *darlingi*-positive water body	Continuous
	Distance to nearest forest	Continuous
	Percent forest cover 2016 (50/100/250/500m buffers)	Continuous
	Percent forest loss 2010–2016 (50/100/250/500m buffers)	Continuous
Aquatic characteristics	Algae	Binary
	Emergent vegetation	Binary
	Floating vegetation	Binary
	Fish	Binary
	Amphibians	Binary
	Other *Nyssorhynchus*, *Stethomyia*, or *Anopheles* spp. larvae	Binary
	Bed material (sand, organic, mixed, or mud)	Discrete
	Water movement	Binary
	Water turbid	Binary
	Water body seasonality (temporal or permanent)	Discrete
	Water body depth	Continuous
	Alkalinity (zero/non-zero)	Binary
	Conductivity	Continuous
	Hardness (zero/non-zero)	Binary
	pH	Continuous
	Salinity	Continuous
	Water temperature	Continuous

To account for the low percentage of missing data in the final dataset (2% overall, 8% or less for each variable), multiple imputation was done using the cart method in the R package ‘mice’ [75]. Five imputations were calculated, with 20 iterations each. The imputed datasets were used to construct logistic mixed-effects models in the R package ‘mitml’ [76], with the presence of each *Nyssorhynchus* species as the outcome and the water body ID as a random intercept to account for multiple collections conducted at each water body. Village was included as a fixed effect and not a higher-level random intercept so that between-village differences in the presence of *Nyssorhynchus* species could be assessed. The relationship between each variable and the presence of each *Nyssorhynchus* species was evaluated using bivariate models. For the population density variables, vegetation and water indices, and forest cover variables, it was unknown which, if any, variable at which radius buffer would affect the presence of the *Nyssorhynchus* species. Bivariate models were built for each variable at each radius, and for each category (vegetation index: EVI and NDVI; population density: distance to nearest inhabited house, number of people in radius, presence/absence of any people in radius; NDWI; percent forest cover; percent forest loss 2010–2016), the variable with the highest bivariate log likelihood was selected to be evaluated for inclusion in the multivariate model. A forward stepwise process was used to build the multivariate model using only variables with bivariate *p*<0.2. Each variable was added in order of its log likelihood in the bivariate model, and a variable was retained if its *p*-value was <0.2 in the final model.

To explore the effect of environmental variables on the overall community assemblage of *Nyssorhynchus* larvae, we used redundancy analysis (RDA). The RDA was computed in the R package ‘vegan’ [77], using the Hellinger-transformed presence/absence matrix for all identified larval species as the community data matrix and the environmental variables listed in Table 2 (with the exception of the presence of other Anophelinae species larvae) as the constraining variables. Missing data in the environmental dataset was imputed using the imputeFAMD function in the R package ‘missMDA’ [78], using five components to predict the missing entries as suggested by the estim_ncpFAMD function. A single variable and buffer size was selected for the vegetation index, NDWI, forest cover, forest loss, and population density variables by computing RDAs using each variable separately and selecting the variable in each group that resulted in the RDA with the highest adjusted R^2^. The RDA biplot was visualized using the R package ‘ggords’ [79].

### Analysis of malaria case data

Malaria case data for all 8 villages from 2016 were obtained from the local health authority (Dirección Regional de Salud Loreto, DIRESA). Where possible, cases were matched to georeferenced houses to correspond with the census data described above. To reduce the likelihood of including duplicate cases, all repeat diagnoses of *P*. *vivax* within 60 days or *P*. *falciparum* within 30 days for the same person were excluded, as in [80]. Since water bodies were sampled only once every 2–4 months, the cases and the presence of *Ny*. *darlingi* larvae were aggregated by 6-month season (rainy season: January-June 2016, dry season: July-December 2016). Separately for each season, the distance from each house to the nearest water body positive for *Ny*. *darlingi* at least once during the season was calculated using the R package ‘sp’ [73] (S4 Dataset). A separate Poisson regression for each season was constructed in R v. 3.5.2 using the log number of people in each house as the offset, the number of malaria cases in each house during the season as the outcome, and the distance to the nearest *Ny*. *darlingi*-positive water body as the explanatory variable. The village of VIB was excluded from the dry season analysis because no *Ny*. *darlingi*-positive water bodies were identified in this village during this season.

### Bacterial 16S rRNA amplification and sequencing

Larvae for bacterial 16S rRNA sequencing were selected from among 3^rd^ and 4^th^ instar larvae collected in LIB, SAL, and URC. Only larvae collected in September and November 2016 (dry season) were included, to minimize any potential seasonal effects on the microbiome. DNA was extracted from whole larvae using the DNeasy Blood & Tissue kit following surface sterilization in 100% ethanol. Prior to bead-beating, larvae were suspended in the manufacturer-recommended enzymatic lysis buffer (containing lysozyme) to enhance DNA purification from gram-positive bacteria as suggested in [81]. All extractions and post-extraction manipulation of samples and 16S PCR reactions were performed in a biosafety cabinet with pre-sterilized materials where possible to avoid contamination, and all extractions were performed using the same extraction kit to avoid batch contamination effects [82]. A negative extraction control with reagents only was processed along with the samples from extraction through sequencing. The larvae were identified to species using ITS2 PCR-RFLP as described above. Following identification, 95 larvae and the negative extraction control were selected for 16S bacterial sequencing (S5 Dataset). These samples include larvae of three species (*Ny*. *darlingi*, *Ny*. *rangeli*, and *Ny*. *triannulatus* s.l.) from 12 water bodies, selected to maximize the number of water bodies with multiple species present.

Bacterial 16S rRNA gene V3-V4 variable regions were amplified using the Illumina adapter overhang-linked primers suggested in the Illumina 16S Metagenomic Sequencing Library Preparation guide ([83], Table I in S1 File). PCR reactions were performed in a 25μl reaction including 5μl extracted DNA, 5μl each 1μM forward and reverse primers, and 10μl 2X KAPA HiFi HotStart ReadyMix. Reaction conditions were as follows: 95°C for 3 minutes; 30 cycles of 95°C for 30s, 55°C for 30s, and 72°C for 30s; and 72°C for 5 minutes. Products were visualized on 1% agarose gels. For samples that did not amplify using the above reaction conditions (n = 18 larvae + extraction control), an identical protocol using a 35-cycle reaction was used. All PCR reactions were performed in triplicate, and pooled products of the triplicate reaction for each sample were sent to the Wadsworth Center Applied Genomic Technologies Core for PCR clean-up, a second PCR to attach dual indices and Illumina sequencing adapters, and sequencing on the Illumina MiSeq system. All reads were deposited in the NCBI Sequence Read Archive (SRA; BioProject ID PRJNA494695).

### Bacterial 16S rRNA sequencing data analysis

The Quantitative Insights Into Microbial Ecology (QIIME) 1.9.1 pipeline [84] multiple_join_paired_ends.py and multiple_split_libraries_fastq.py scripts were used to prepare the sequencing reads for analysis. The QIIME 1.9.1 pick_open_reference_otus.py script, which wraps uclust for clustering [85], PyNAST for alignment [86], RDP Classifier for assigning taxonomy [87], and FastTree for building a phylogenetic tree [88], was used to assign 16S rRNA reads to operational taxonomic units (OTUs). Reverse strand matching was enabled in uclust, OTUs were matched to the SILVA 128 rRNA database [89] at a 97% identity threshold, alignments were filtered using an allowed gap fraction of 0.8 and an entropy threshold of 0.1, and lane mask filtering was suppressed. A single OTU (an uncultured *Delftia* spp., GU731299) had a higher relative abundance in the negative control than in any larva (control:maximum larvae relative abundance ratio = 6.38 vs. <0.5 for all other OTUs). This OTU accounted for 38% of the 3704 identified reads from the negative control (the next most prevalent OTU accounted for only 7% of the reads) and 1.5% of larval reads and was excluded for all downstream analyses. In addition, non-bacterial OTUs (n = 2) and low-abundance OTUs accounting for <0.1% of reads were filtered from the final table. The final OTU table is included as S6 Dataset.

The QIIME 1.9.1 summarize_taxa_through_plots.py script was used to visualize differences in bacterial composition across samples and groups. To compare the beta diversity across groups of samples, the QIIME 1.9.1 beta_diversity_through_plots.py script was used to rarefy the OTU table to 13,000 sequences per individual (rarefaction curves indicated that the alpha diversity saturated at about 10,000 sequences (Fig IV in S1 File)), and to compute unweighted and weighted UniFrac [90] and Bray-Curtis distance matrices, as well as a principal coordinates analysis (PCoA) for each distance matrix. Taxonomic composition and principal coordinates analysis plots were created in R using the ‘ggplot2’ package [91]. QIIME 2 v. 2017.12 was used to compute pairwise analyses of similarities (ANOSIMs) of the beta diversity distance matrices (beta-group-significance), alpha rarefaction curves (alpha-rarefaction), and an analysis of composition of microbes (ANCOM; qiime composition ancom) [92].

## Results

### Ecological characterization of Anophelinae larval habitats

**Fig 2:**
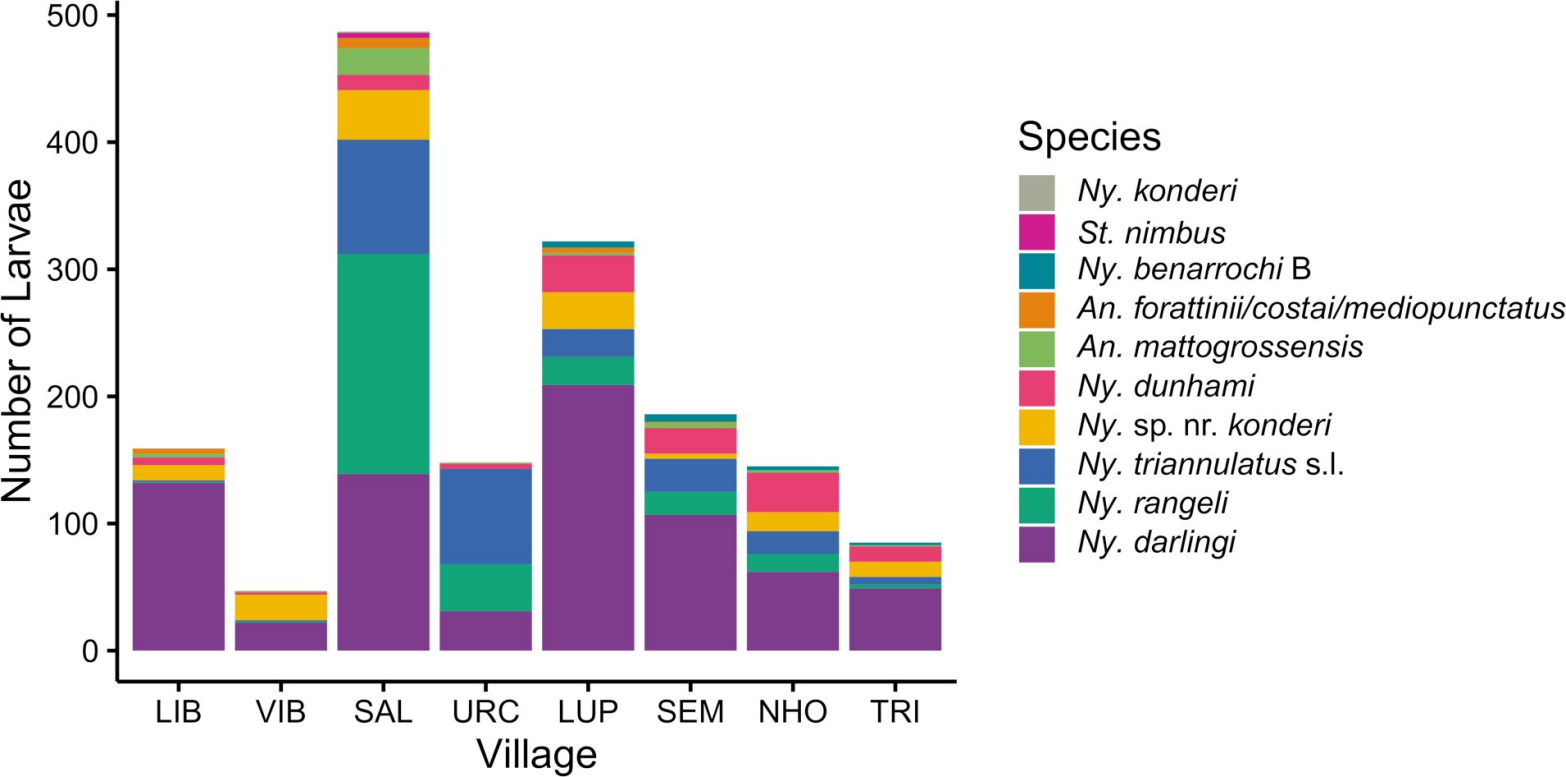
Number and species of identified Anophelinae larvae collected in each village, January 2016-March 2017.

A total of 1579 larvae identified as *Nyssorhynchus*, *Anopheles*, or *Stethomyia* species were collected in 88 water bodies in the 8 villages between January 2016 and March 2017 (S1 Dataset). This excludes 102 larvae lost in processing, 32 larvae for which the *COI* product did not amplify, and 24 larvae identified as non-*Anopheles*, *Kerteszia*, *Lophopodomyia*, *Nyssorhynchus*, or *Stethomyia* species. The most commonly identified species was *Ny*. *darlingi* (n = 751), followed by *Ny*. *rangeli* (n = 269), *Ny*. *triannulatus* s.l. (n = 239), *Ny*. sp. nr. *konderi* (n = 131), *Ny*. *dunhami* (n = 116), *Anopheles mattogrossensis* Lutz & Neiva (n = 35), *Anopheles forattinii* Wilkerson & Sallum/*Anopheles costai* Fonseca & Ramos/*Anopheles mediopunctatus* Lutz (n = 17) (these three species are closely related [93] and could not be differentiated by *COI* barcode sequences), *Nyssorhynchus benarrochi* B Ruiz (formerly *Anopheles benarrochi* B [5]) (n = 16), *Stethomyia nimbus* Theobald (formerly *Anopheles nimbus* [5]) (n = 4), and *Ny*. *konderi* (n = 1). Each of the six most common species was collected in all 8 villages (with the exception of *Ny*. sp. nr. *konderi* in URC). *Ny*. *benarrochi* B was only collected in the four villages to the south and west of Iquitos (LUP, SEM, NHO, and TRI), and *St*. *nimbus* and *Ny*. *konderi* were only collected in SAL (Fig 2). The species composition varied by village (Fig 2) but was relatively consistent over time within each village (Fig II in S1 File).

*Nyssorhynchus darlingi*, *Ny*. *rangeli*, *Ny*. *triannulatus* s.l., and *Ny*. sp. nr. *konderi* were all collected from both artificial and natural water bodies (Table 3). All fish ponds that were active throughout the study period (n = 17) were positive for *Ny*. *darlingi* at least once.

**Table 3:** Types of water bodies in peri-Iquitos sampled for Anopheline larvae and positive for *Ny*. *darlingi*, *Ny*. *rangeli*, *Ny*. *triannulatus* s.l., and *Ny*. sp. nr *konderi* at least once during 2016–2017 collections.

Water Body Type	Total water bodies sampled	Number (%) positive for *Ny*. *darlingi*	Number (%) positive for *Ny*. *rangeli*	Number (%) positive for *Ny*. *triannulatus* s.l.	Number (%) positive for *Ny*. sp. nr. *konderi*
Abandoned fish pond^1^	5	2 (40%)	1 (20%)	1 (20%)	1 (20%)
Active fish pond^1^	17	17 (100%)	13 (76%)	14 (82%)	12 (71%)
Active ➔ abandoned fish pond^2^	5	4 (80%)	3 (60%)	4 (80%)	2 (40%)
Pond	5	4 (80%)	2 (40%)	2 (40%)	2 (40%)
Pool	10	1 (10%)	0 (0%)	1 (10%)	1 (10%)
Stream/river	41	26 (63%)	14 (34%)	13 (32%)	13 (32%)
Swamp	5	5 (100%)	3 (60%)	3 (60%)	3 (60%)
**Total**	88	59 (67%)	36 (41%)	38 (43%)	34 (39%)

^1^Abandoned/active for duration of study

^2^Active at first collection, but abandoned over the course of the study

The final analysis dataset included 403 collection points for 88 water bodies sampled across 5–6 collections, excluding 84 collection points at which the water body was dry. Anophelinae species were often found co-occurring at collection points; at least two species were present in 138 (70%) of 197 collection points that had any species present, and in one case, eight species were present at the same collection point. Affinity indices calculated between pairs of the five most common species indicated that two pairs of species showed an affinity (affinity index>0.5): *Ny*. *darlingi* and *Ny*. *rangeli*; and *Ny*. *rangeli* and *Ny*. *triannulatus* s.l. (Table 4).

**Table 4:** Affinities between 5 most commonly identified larval species. The number of co-occurrences of species in the same water body at the same collection are shown above the diagonal, and Fager’s affinity index is shown below the diagonal. Affinity indices >0.5 (indicative of affinity) are bolded.

	*Ny*. *darlingi*	*Ny*. *dunhami*	*Ny*. *rangeli*	*Ny*. *triannulatus* s.l.	*Ny*. sp. nr. *konderi*
***Ny***. ***darlingi***	-	57	62	54	53
***Ny***. ***dunhami***	0.49	-	22	18	28
***Ny***. ***rangeli***	**0.55**	0.27	-	42	19
***Ny***. ***triannulatus* s.l.**	0.48	0.21	**0.58**	-	15
***Ny***. **sp. nr *konderi***	0.48	0.37	0.24	0.18	-

**Fig 3:**
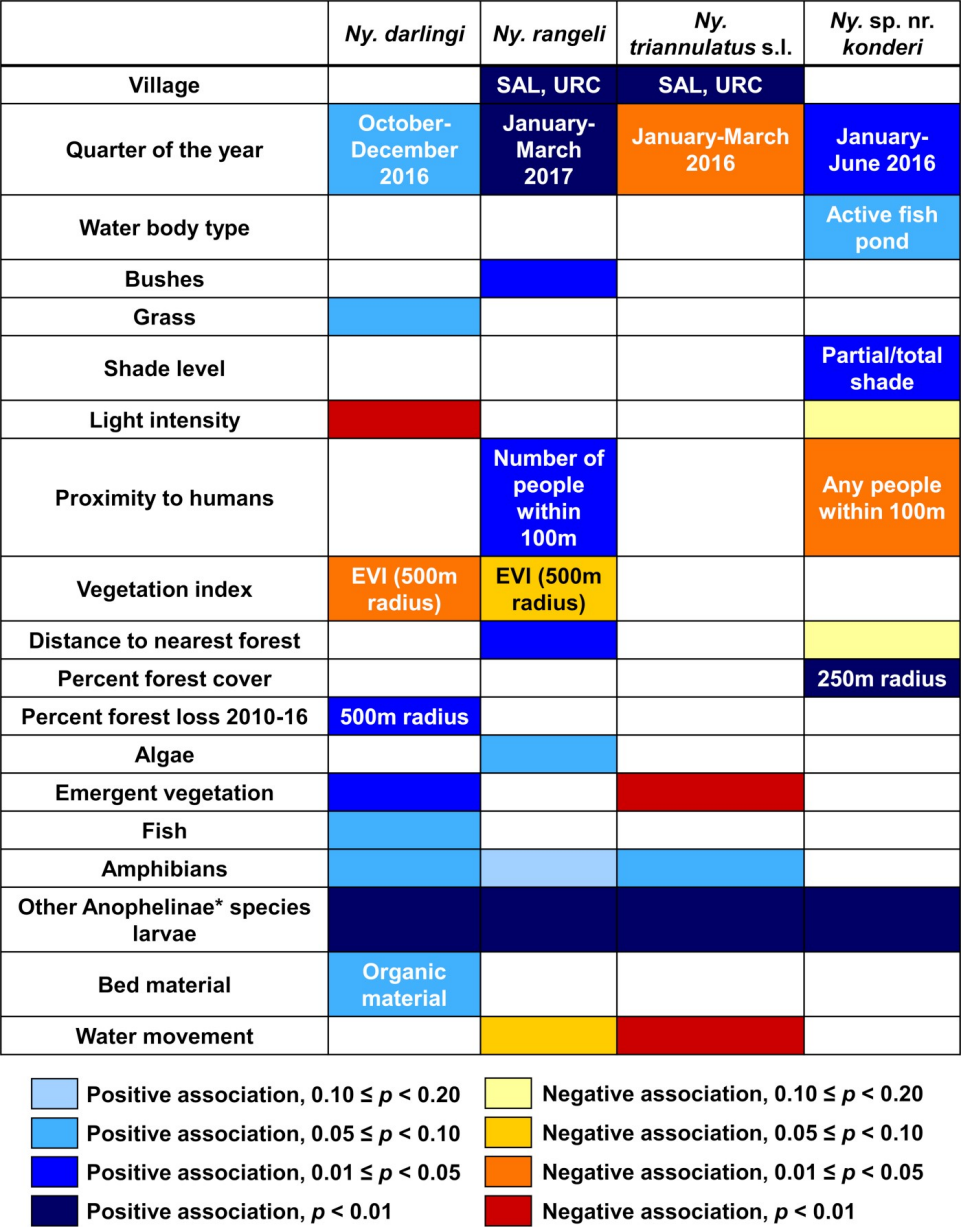
Heat map of results of multivariate logistic mixed-effects models for the presence of *Ny*. *darlingi*, *Ny*. *rangeli*, *Ny*. *triannulatus* s.l., and *Ny*. sp. nr. *konderi* larvae. Four shades of blue coloring represent positive associations between the variable and the presence of each species, and yellow, orange, or red coloring each represent a negative association, with more intense colors representing lower *p*-values for the coefficient in the regression model. White coloring indicates that the variable was not included in the multivariate model for that species. *Includes any identified larvae in the genera *Nyssorhynchus*, *Anopheles*, or *Stethomyia*.

Of the 403 collection points, *Ny*. *darlingi* was present in 169 (42%), *Ny*. *rangeli* in 66 (16%), *Ny*. *triannulatus* s.l. in 65 (16%), and *Ny*. sp. nr. *konderi* in 61 (15%). In multivariate logistic mixed-effects models, the presence of all four species was most strongly associated with the presence of other *Nyssorynchus*, *Anopheles*, or *Stethomyia* spp. larvae (OR>14, *p*<0.001 for all four species; Fig 3; Tables III-VI in S1 File). In addition, the presence of *Ny*. *darlingi* was significantly positively associated with the presence of emergent vegetation (OR = 2.54, *p* = 0.036) and percent forest loss between 2010 and 2016 at a 500m radius (OR = 1.38, *p* = 0.016), and significantly negatively associated with EVI at a 500m radius (OR = 0.01, *p* = 0.014) and light intensity (OR = 0.14, *p* = 0.007). The odds of *Ny*. *darlingi* presence differed significantly among villages in the bivariate, but not the multivariate model (Table III in S1 File). *Ny*. *darlingi* presence was not significantly associated with highway vs. riverine habitat (riverine OR = 1.72 vs. highway, *p* = 0.335).

The presence of *Ny*. *rangeli* was significantly positively associated with the villages SAL and URC (OR = 6.37 vs. the other 6 villages, *p* = 0.002), the presence of bushes (OR = 2.92, *p* = 0.018), the distance to the nearest forest (OR = 1.01, *p* = 0.017), the number of people living in a 100m radius (OR = 1.02, *p* = 0.013), and the January-March 2017 quarter (OR = 8.17 vs. July-September 2016, *p* = 0.004) in the multivariate model (Table IV in S1 File).

The presence of *Ny*. *triannulatus* s.l. was significantly positively associated with the same villages as *Ny*. *rangeli*: SAL and URC (OR = 16.45 vs. the other 6 villages, *p*<0.001), and significantly negatively associated with the presence of water movement (OR = 0.07, *p* = 0.002), the presence of emergent vegetation (OR = 0.15, *p* = 0.004), and the January-March 2016 quarter (OR of all other quarters>8, *p*≤0.034) in the multivariate model (Table V in S1 File).

The presence of *Ny*. sp. nr. *konderi* was significantly positively associated with partial and total shade (OR = 10.02/12.57 vs. no shade and *p* = 0.016/0.010, respectively), the percent forest cover in a 250m radius (OR = 1.06, *p* = 0.001), and the January-March and April-June 2016 quarters (OR = 4.52/4.05 vs. January-March 2017 and *p* = 0.013/0.023, respectively), and significantly negatively associated with the presence of any people living in a 100m radius (OR = 0.32, *p* = 0.014) in the multivariate model (Table VI in S1 File).

The RDA results were consistent with the results of the multivariate logistic mixed-effects models, highlighting, for example, the association of *Ny*. *darlingi*-positive habitats with emergent vegetation and recent forest loss (Fig III in S1 File). The RDA also emphasizes the ecological similarity of *Ny*. *triannulatus* s.l. and *Ny*. *rangeli* habitats, and of *Ny*. sp. nr. *konderi* and *Ny*. *dunhami* habitats.

### Houses with more malaria cases are closer to *Ny*. *darlingi* larval habitats

**Fig 4:**
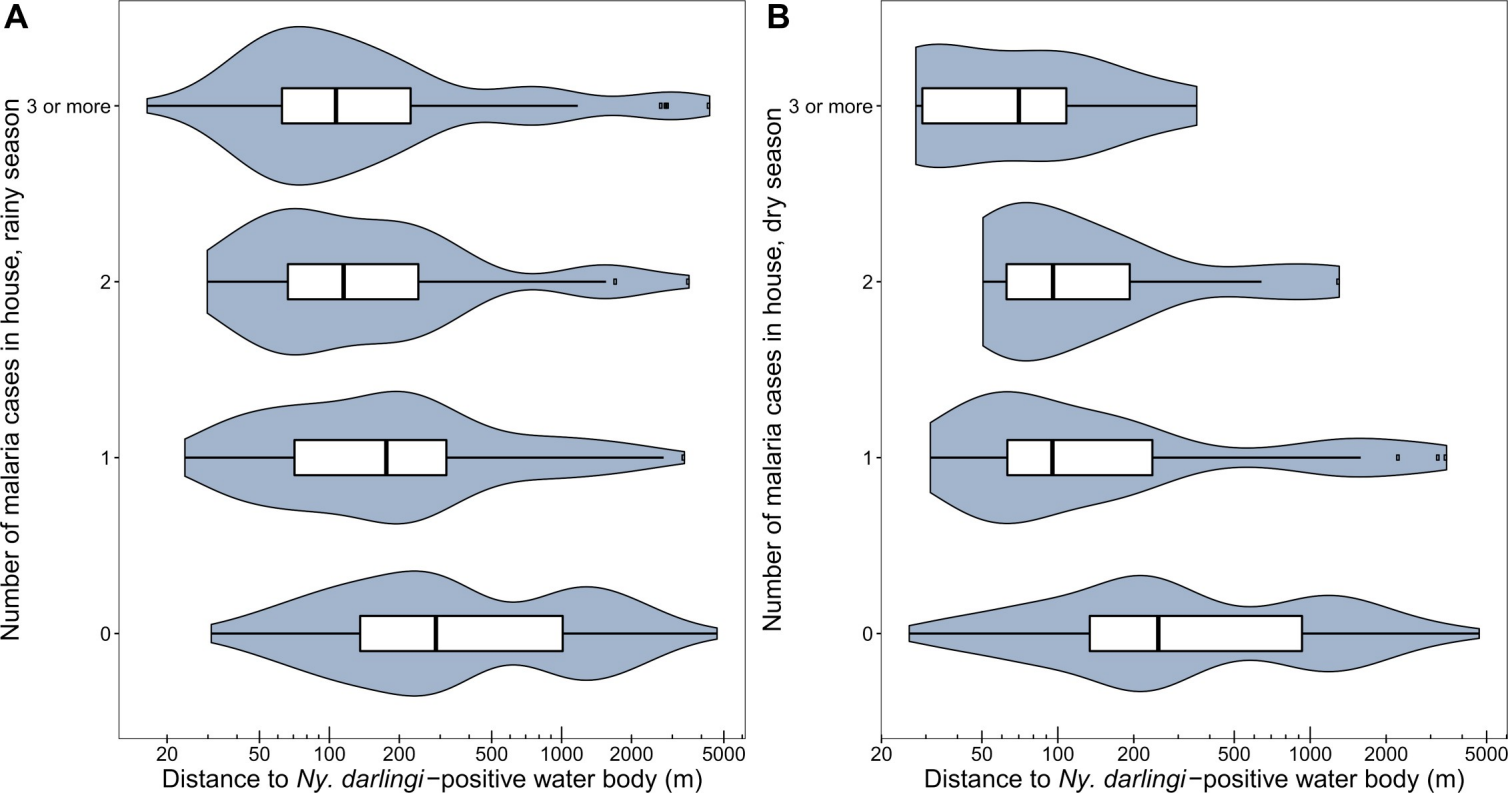
Houses with more diagnosed malaria cases are closer to *Ny*. *darlingi*-positive water bodies. Includes malaria cases diagnosed in 2016 in each georeferenced house in the eight study villages in the (A) rainy (January-June) and (B) dry (July-December) seasons. *Ny*. *darlingi*-positive water bodies include all water bodies in which *Ny*. *darlingi* was collected at least once during the season indicated. The minimum distance to a *Ny*. *darlingi*-positive water body is shown on a log scale.

A total of 556 malaria cases (367 (66%) *P*. *vivax*, 189 (34%) *P*. *falciparum*) were reported in the 8 study villages during 2016, excluding 22 repeat diagnoses. The Annual Parasite Index (API) for each village in 2016 ranged from 22 (URC) to 659 (LUP) (Table VII in S1 File). The final analysis dataset consisted of 442 houses from the 8 villages with a combined 1951 inhabitants and 498 malaria cases, excluding 18 inhabitants and 53 malaria cases that were unable to be linked to a georeferenced house, and 5 cases reported from VIB in the dry season excluded because no *Ny*. *darlingi*-positive water body was identified in this village during this season (S4 Dataset). The number of malaria cases in each house was negatively associated with the distance to the nearest water body positive for *Ny*. *darlingi* in the rainy season (Poisson rate ratio per 100m distance = 0.98, 95% CI 0.96–0.996, *p* = 0.02) and in the dry season (Poisson rate ratio per 100m distance = 0.91, 95% CI 0.86–0.95, *p* = 0.0005) (Fig 4).

### Bacterial composition varies among larval species

Sequencing of 16S rRNA amplicons resulted in a total of 18,427,247 paired-end reads from 95 larvae, with a median of 166,453 reads per larva (range: 55,335–719,042), and 5,865 reads from the negative extraction control. After OTU picking and filtering, the final OTU table for the 95 larvae consisted of 12,392,204 reads matched to 89 bacterial OTUs (S5 and S6 Datasets).

**Fig 5:**
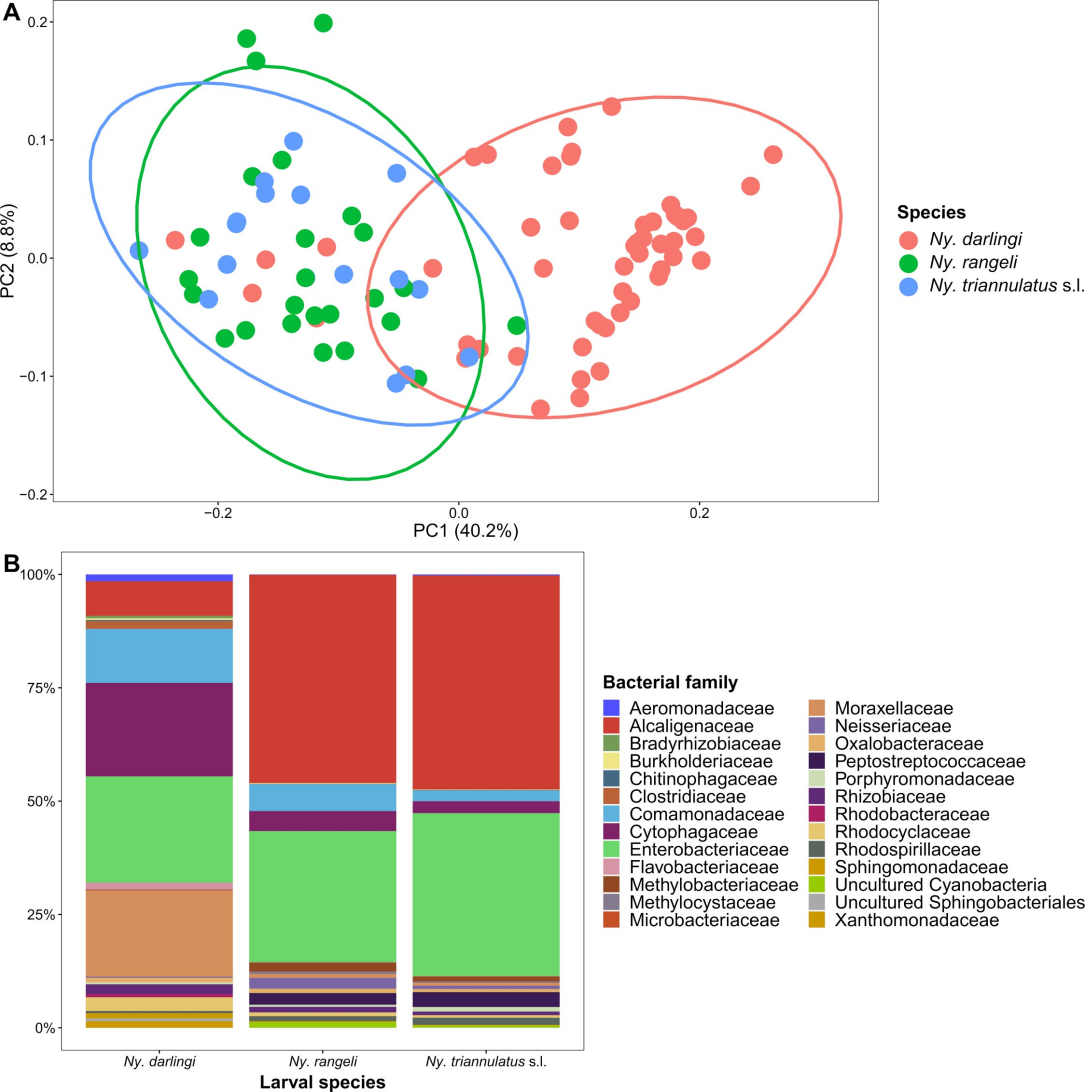
Bacterial composition of *Nyssorhynchus* larvae differs by species. (A) Principal Coordinates Analysis (PCoA) of unweighted UniFrac distances of larval bacterial communities. Individual larvae are colored by species, with ellipses indicating 95% confidence intervals around each species. (B) Pooled bacterial family composition for each larval species.

A principal coordinates analysis (PCoA) of the unweighted Unifrac distance matrix indicated that the samples clustered by larval species (Fig 5A), with the *Ny*. *darlingi* larvae clustering together, and apart from the *Ny*. *rangeli* and *Ny*. *triannulatus* s.l. larvae. PCoAs of the weighted Unifrac and Bray-Curtis distance matrices showed similar clustering (Fig V in S1 File). The samples did not cluster by water body (Fig VI in S1 File). An ANOSIM of the unweighted Unifrac distance matrix indicated that the bacterial composition was more similar among larvae from the same species (overall R = 0.55, *p* = 0.001; *Ny*. *darlingi* vs. *Ny*. *rangeli* R = 0.61, *p* = 0.001; *Ny*. *darlingi* vs. *Ny*. *triannulatus* s.l. R = 0.63, *p* = 0.001; *Ny*. *rangeli* vs. *Ny*. *triannulatus* s.l. R = 0.07, *p* = 0.096) than among larvae from the same water body (overall R = 0.12, *p* = 0.001) or village (overall R = 0.08, *p* = 0.03). Measured ecological variables of the water bodies, including presence of vegetation, water chemistry measurements, and vegetation and water indices (S5 Dataset) were also tested for their effect on the bacterial composition; none had as strong as an effect as larval species (next highest ANOSIM R = 0.25).

The most abundant bacterial families identified from *Ny*. *darlingi* larvae were Enterobacteriaceae, Cytophagaceae, and Moraxellaceae; from *Ny*. *rangeli* and *Ny*. *triannulatus* s.l. larvae, the most abundant were Alcaligenaceae and Enterobacteriaceae (Fig 5B, Fig VII in S1 File). By ANCOM, 33 of the 89 OTUs were differentially abundant comparing *Ny*. *darlingi* to *Ny*. *rangeli* and *Ny*. *triannulatus* s.l. These included three OTUs in the family Cytophagaceae (all in the genus *Flectobacillus*) that were more abundant in *Ny*. *darlingi*; four OTUs in the family Alcaligenaceae (in the genera *Bordetella*, *Candidimonas*, *Castellaniella*, and *Pusillimonas*) that were more abundant in *Ny*. *rangeli* and *Ny*. *triannulatus* s.l.; and six OTUs in the family Enterobacteriaceae (all in the genus *Thorsellia*), two of which were more abundant in *Ny*. *darlingi* and four of which were more abundant in *Ny*. *rangeli* and *Ny*. *triannulatus* s.l. (S1 Table).

## Discussion

In this study, we investigate the larval ecology of *Ny*. *darlingi* in the peri-Iquitos region of Amazonian Peru in the context of human landscape modification and malaria risk. Furthermore, we provide evidence that *Nyssorhynchus* species from the same larval habitats have distinct microbiomes. This study represents, to our knowledge, the first published characterization of *Ny*. *darlingi* larval habitats in the peri-Iquitos region since 2001 [20], and the first that has included riverine villages. The epidemiology of malaria in the Loreto Department has changed significantly since 2001; a comprehensive malaria control program resulted in a dramatic drop in malaria incidence between 2005 and 2011, followed by a steady increase in the number of overall malaria cases and the proportion caused by *P*. *falciparum* since then [16]. This has been accompanied by behavioral [9] and genetic [21] changes in the adult *Ny*. *darlingi* population in peri-Iquitos in the same timeframe. This study provides an updated understanding of the ecology of the primary malaria vector in the region, as well as possible secondary vectors, which will fundamentally inform integrated vector control methods, including targeted larval source management.

In this study, *Ny*. *darlingi* were more likely to be present in water bodies in areas with a higher amount of recent deforestation (a higher percent forest loss in a 500m radius between 2010 and 2016) and a lower vegetation index (a lower EVI at a 500m radius). These associations are consistent with the results of Vittor et al.’s study in the peri-Iquitos region [20], that found that increased forest cover in a 1x1km grid decreased the probability of *Ny*. *darlingi* larval presence. The relationship between deforestation and both *Ny*. *darlingi* habitat suitability and overall malaria transmission has not been clearly defined, mainly due to differences in the definition of deforestation in different studies [94]. In the Brazilian Amazon, the forest fringe hypothesis has been proposed, whereby malaria risk is highest at the edges of deforested areas [95], particularly in small deforested patches [96]. In this transition zone between forested and deforested areas, vectors have ample access to human blood meals, but also to shaded water bodies [22, 24]. In the Peruvian Amazon, the forest cover level is overall much higher than it is in the Brazilian Amazon [94]; for the current study, the forest cover in a 500m radius around the sampled water bodies ranged from 33% to 89%. It is possible that deforestation differentially affects vector populations and overall malaria risk at different forest cover levels [94]; perhaps the water bodies in deforested landscapes in the peri-Iquitos region act comparably to the forest fringes in Brazil. Clearly, more research on the dynamics between deforestation and malaria transmission across the Amazon is necessary.

*Nyssorhynchus darlingi* larval habitats have also been found in previous studies to be associated with human presence directly [20, 22, 23], and indirectly via aquaculture; fish ponds have been implicated as *Ny*. *darlingi* larval habitats [20, 23, 25] and have been hypothesized to increase the risk of malaria transmission in the Peruvian and Brazilian Amazon [26, 97]. While we did not see a significant association between the presence of *Ny*. *darlingi* and the number of people living in a 100m radius (OR = 1.02, *p* = 0.09), a significant increase in the odds of *Ny*. *darlingi* presence in active fish ponds (OR = 3.24, *p* = 0.03) was observed in bivariate models (Table III in S1 File). Neither variable was included in the final multivariate model. Active fish ponds were consistently positive for multiple Anophelinae species characterized in this study, including *Ny*. *darlingi*. However, the five most abundant species were also frequently collected from natural water bodies, particularly streams and rivers, highlighting the need for larval control strategies to target natural as well as artificial water bodies.

The current study identified emergent vegetation and lower light intensity as predictive for *Ny*. *darlingi*. Associations between *Ny*. *darlingi* larval habitats and various types of vegetation have been reported previously, including algae [20, 98], grassy and floating vegetation [54, 99], patches of detritus [22, 100–102], and submerged vegetation [103, 104]. Vegetation cover in and around larval habitats could provide food for larvae, shelter from predators, and favorable oviposition conditions for adults [14]. An association between lower light intensity and the presence of *Ny*. *darlingi* has been reported previously in Brazil [22], and *Ny*. *darlingi* have been consistently reported to oviposit in shaded or partially shaded habitats [54, 99, 102, 103]. This association could represent a direct effect of light exposure or temperature on development of the larvae, or an indirect effect on food sources or habitat stability [14].

This study was conducted in villages with very high rates of malaria transmission (Table VII in S1 File). We found that houses in the eight study villages that were located closer to *Ny*. *darlingi*-positive water bodies had more cases of malaria in both the rainy and dry seasons. Proximity to *Ny*. *darlingi* larval habitats has been identified as a malaria risk factor previously in the Amazon [24, 97, 105]. While it is clear that not all cases of malaria are acquired at home [50], this association indicates that larval source management targeting water bodies near villages could be employed as part of an integrated intervention strategy to reduce malaria risk in the peri-Iquitos region.

The most significant predictor for the presence of all four larval species characterized in this study was the presence of other Anophelinae species in the same water body. In addition, high affinity indices were seen between *Ny*. *darlingi* and *Ny*. *rangeli*, and between *Ny*. *triannulatus* s.l. and *Ny*. *rangeli*. This could indicate that the Anophelinae species in this study have similar requirements for larval habitats, or that the larvae interact synergistically in these water bodies. Similar affinities have been identified previously between *Nyssorhynchus* species [106–108], including between *Ny*. *darlingi* and *Ny*. *triannulatus* s.l. in Venezuela [109]. The co-occurrence of these putative vector species could simplify larval source management control strategies, as multiple species could be targeted in a single water body.

Though *Ny*. *darlingi*, *Ny*. *rangeli*, and *Ny*. *triannulatus* s.l. were found to co-occur often in larval habitats, we found differences in the larval microbiome between *Ny*. *darlingi* and the other two species collected from the same water bodies. This could reflect different niches occupied by each species within the same water body, leading to differences in the composition of bacterial species to which each mosquito species is exposed. Alternatively, there could be underlying biological differences in the microbiome among larval species [110]. The redundancy analysis results, which show that *Ny*. *rangeli* and *Ny*. *triannulatus* s.l. larvae were collected in ecologically similar habitats (Fig III in S1 File), provide support for the first possibility.

Our results contrast with Bascuñán et al.’s recent study characterizing the bacterial composition of *Nyssorhynchus* species, that found no differences between *Ny*. *darlingi* and *Ny*. *nuneztovari* s.s. adults collected from coastal Colombia [36]. It is possible that the microbiomes of *Ny*. *darlingi* and *Ny*. *nuneztovari* s.s. are more similar than those of the species in our study, or that species differences in the *Nyssorhynchus* microbiome occur in larvae, but not in adults. As it is clear that there is a shift in microbiome composition after adult emergence in African [27, 38, 111] and Neotropical [36] malaria vectors, future studies could determine whether the species difference that we identified is maintained in adult *Nyssorhynchus* mosquitoes. Previous research in other genera has found a greater impact of larval habitat than species on the mosquito larval microbiome [38–40]. However, these studies collected larvae from small water bodies, such as containers [39] and irrigation ditches/puddles [38, 40]. All larvae included for bacterial 16S rRNA sequencing in the present study were collected from fish ponds or streams; larvae could be exposed to a wider variety of bacteria in these larger water bodies, which could explain the lack of a larval habitat-specific bacterial signature.

Our study adds to an increasing literature characterizing the microbiome of Neotropical malaria vectors. There are overlaps between the bacterial composition of *Ny*. *darlingi* larvae in the present study and that reported in *Ny*. *darlingi* adults in past studies, including the presence of bacteria in the families Enterobacteriaceae [33, 35, 36], Moraxellaceae [35, 36], Aeromonadaceae [36], Rhodocyclaceae [36], and Comamonadaceae [36]. Furthermore, our identification of *Thorsellia* spp. bacteria in *Ny*. *darlingi* is consistent with the recent description of a new species of bacteria, closely related to *Thorsellia*, isolated from *Ny*. *darlingi* larvae [34].

Both *Ny*. *rangeli* [54, 108, 112] and *Ny*. *triannulatus* s.l. [113] have been described as habitat generalists; this is consistent with the lack of significant environmental predictors for the presence of each species found in the current study. We did find *Ny*. *rangeli* to be associated with water bodies with bushes nearby that were located farther from forests and closer to human habitations. Larvae of this species was previously collected from an eutrophized dam in Rio de Janeiro State, Brazil [114]; the current study further confirms the tendency of *Ny*. *rangeli* to oviposit in human-associated habitats. The association we detected between *Ny*. *triannulatus* s.l. and non-moving water is consistent with previous studies that found associations of this species complex with large, permanent water bodies such as lagoons and fish ponds, while the negative association we found between *Ny*. *triannulatus* s.l. and emergent vegetation contrasts with previous studies that associated this species with vegetation [104, 115–118].

From the Oswaldoi-Konderi complex, we report the presence of both *Ny*. sp. nr. *konderi* (which has previously been collected in Loreto [61]) and a single specimen of *Ny*. *konderi* in our study villages. The association we identified between *Ny*. sp. nr. *konderi* and shaded water bodies has been previously reported for members of the Oswaldoi-Konderi species complex in Suriname [99]; however, a recent report from the Brazilian Amazon found *Ny*. *oswaldoi* s.l. habitats associated with more sun exposure [104]. We also found an association between *Ny*. sp. nr. *konderi* presence and habitats with a higher forest cover and further from human habitations. This association has not been previously reported for Oswaldoi-Konderi species complex members, though previous studies have found *Ny*. *oswaldoi* s.l. associated with roads [54, 119]. It is possible that different species in this species complex have different larval habitat preferences. Incorporating molecular identification methods into future Anophelinae larval studies in the Neotropics could help to resolve these inconsistencies.

This study had several limitations. First, it was a relatively short-term exploratory study conducted in the absence of recent baseline data of the larval ecology of malaria vectors in the peri-Iquitos region. There is a need for more longitudinal sampling in this region so that larval habitats can be more thoroughly characterized and seasonal differences explored. Second, we sampled only the water bodies we were able to identify by satellite imagery and ground-truthing within a 1km radius of each village. Future studies using more sophisticated technology such as drones [120] could help identify additional water bodies. Third, our larval dipping methods, though standard, may not have captured the entire species diversity in each water body in each collection. Neotropical Anophelinae larvae have been successfully collected using methods that sample the interior of large water bodies in addition to the perimeter [104], perhaps providing a more complete sampling of the larvae present. However, of the larvae that we collected, we were able to identify over 90% using a combination of morphological and molecular methods. Fourth, malaria cases included in this study were obtained from passive case reporting using health post data, which likely underestimates the overall malaria burden in these villages. Fifth, our larval microbiome analysis is limited by the small sample size and number of water bodies and species represented. Future studies including more comprehensive sampling could help to confirm the species differences we identified. Furthermore, the use of alternative analysis techniques, such as the use of exact sequence variants rather than OTUs [121], should be explored.

### Conclusions

In this longitudinal study, we described ecological characteristics of the larval habitats of Anophelinae malaria vectors in eight villages on four river systems and a highway in the peri-Iquitos region. *Nyssorhynchus darlingi*, the primary regional malaria vector, was collected in both natural and artificial water bodies in all eight villages throughout the fifteen-month study period. *Nyssorhynchus darlingi*-positive water bodies were associated with more recent deforestation, a lower vegetation index, lower light intensity, and emergent vegetation, as well as the presence of other Anophelinae species. Despite the high co-occurrence of Anophelinae species in water bodies, we found that *Ny*. *darlingi* larvae had a distinct microbiome compared with *Ny*. *rangeli* and *Ny*. *triannulatus* s.l. larvae. Houses in the study area with more malaria cases were located closer to identified *Ny*. *darlingi* larval habitats. Our findings highlight the potential for larval source management to be a successful control measure in the peri-Iquitos region, as well as the continuing need to better understand the larval ecology of malaria vectors in the heterogeneous Amazon basin landscape so that these control efforts can be more efficiently targeted to reduce the risk of malaria.

## Supporting information

S1 File Contains Tables I-VII and Figures I-VII. Table I: Primer sequences.
•   Table II: Dates of Google Earth imagery used for distance to nearest forest calculations.
•   Table III: Results of bivariate and multivariate logistic mixed-effects models for the presence of Ny. darlingi larvae.
•   Table IV: Results of bivariate and multivariate logistic mixed-effects models for the presence of Ny. rangeli larvae.
•   Table V: Results of bivariate and multivariate logistic mixed-effects models for the presence of Ny. triannulatus s.l. larvae.
•   Table VI: Results of bivariate and multivariate logistic mixed-effects models for the presence of Ny. sp. nr. konderi larvae.
•   Table VII: Number of reported cases of Plasmodium vivax and Plasmodium falciparum in each study village by season, 2016.
•   Fig I: Median-joining COI haplotype network for members of the Nyssorhynchus Oswaldoi-Konderi complex.
•   Fig II: Number and species of identified Anophelinae larvae by quarter in each village.
•   Fig III: Redundancy analysis (RDA) biplot showing the distribution of Nyssorhynchus species larvae in relation to environmental variables.
•   Fig IV: Alpha rarefaction curve from bacterial 16S rRNA sequencing.
•   Fig V: Principal Coordinates Analysis (PCoA) of (A) weighted UniFrac, (B) Bray-Curtis distance matrices of Nyssorhynchus larval bacterial communities, with individual larvae colored by species.
•   Fig VI: Principal Coordinates Analysis (PCoA) of (A) unweighted UniFrac, (B) weighted UniFrac, (C) Bray-Curtis distance matrices of Nyssorhynchus larval bacterial communities, with individual larvae colored by water body.
•   Fig VII: Bacterial family composition of individual Nyssorhynchus larva.
(PDF)

S1 Table Results of Analysis of Composition of Microbiomes (ANCOM), comparing the bacterial OTU composition between *Ny*. *darlingi* and *Ny*. *rangeli*/*Ny*. *triannulatus* s.l. larvae. (CSV)

S1 Dataset All identified *Nyssorhynchus*, *Anopheles*, and *Stethomyia* larvae, with village, coordinates, and date of collection; identification method; species; and GenBank accession number where applicable. (XLSX)

S2 Dataset List of haplotypes of Oswaldoi-Konderi complex members from this study and GenBank displayed in Fig I in S1 File. (XLSX)

S3 Dataset Dataset used for mixed-effects logistic regressions, with one row for each water body at each collection. A data dictionary is included in the second sheet.
(XLSX)

S4 Dataset Dataset used for malaria case data analysis, including an ID number for each georeferenced house in the 8 study villages, the number of censused inhabitants, the season (rainy season = January-June 2016, dry season = July-December 2016), the number of malaria cases diagnosed among inhabitants of each house during the indicated season, and the distance from each house to the nearest water body positive at least once during the indicated season for *Ny*. *darlingi* larvae. (CSV)

S5 Dataset All *Ny*. *darlingi*, *Ny*. *rangeli*, and *Ny*. *triannulatus* s.l. larvae selected for bacterial 16S rRNA sequencing, including the SRA accession number, number of sequenced reads and number of reads in final OTU table, and water body environmental data. (CSV)

S6 Dataset Final OTU table used for QIIME analyses, showing the number of reads corresponding to each bacterial OTU sequenced from each larva. (TXT)
