# Supplementary material for: Malaria vector species in Amazonian Peru co-occur in larval habitats but have distinct larval microbial communities
Source: PLoS Negl Trop Dis. 2019 May 15;13(5):e0007412. doi: 10.1371/journal.pntd.0007412 (PMC6538195; doi:10.1371/journal.pntd.0007412)

S1 File: Supplementary Tables and Figures

Malaria vector species in Amazonian Peru co-occur in larval habitats but have distinct larval microbial communities

Catharine Prussing, Marlon P. Saavedra, Sara A. Bickersmith, Freddy Alava, Mitchel Guzmán, Edgar Manrique, Gabriel Carrasco-Escobar, Marta Moreno, Dionicia Gamboa, Joseph M. Vinetz, Jan E. Conn

Table I: Primer sequences

Use	Primer name	Sequence
ITS2 PCR-RFLP	CP16	5'-GCGGGTACCATGCTTAAATTTAGGGGGTA-3'
ITS2 PCR-RFLP	CP17	5'-GCGCCGCGGTGTGAACTGCAGGACACATG-3'
COI barcode sequencing	LCO1490	5'-GGTCAACAAATCATAAAGATATTGG-3'
COI barcode sequencing	HCO2198	5'-TAAACTTCAGGGTGACCAAAAAATCA-3'
Bacterial 16S rRNA sequencing	Illumina_ Bakt341F	5'-TCGTCGGCAGCGTCAGATGTGTATAAGAGACAGCCT ACGGGNGGCWGCAG-3'
Bacterial 16S rRNA sequencing	Illumina_ Bakt805R	5'-GTCTCGTGGGCTCGGAGATGTGTATAAGAGACAGGA CTACHVGGGTATCTAATCC-3'

Table II: Dates of Google Earth imagery used for distance to nearest forest calculations

Village	Month
Visto Bueno (VIB)	September 2016
Urco Miraño (URC)	September 2016
Salvador (SAL)	September 2016
Libertad (LIB)	September 2016
Lupuna (LUP)	February 2017
Nuevo Horizonte (NHO)	November 2013
El Triunfo (TRI)	November 2013
Santa Emilia (SEM)	N/A – imagery not available for this village

Table III: Results of bivariate and multivariate logistic mixed-effects models for the presence of *Ny. darlingi* larvae

Variable*	Bivariate Odds Ratio (95% CI)	Bivariate *p*-value	Multivariate Odds Ratio (95% CI)	Multivariate *p*-value
**Fixed effects**				
Intercept	-	-	0.14 (0.008, 2.37)	0.171
Non-*Ny. darlingi* Anophelinae species present	35.90 (17.04, 75.63)	<0.001	36.59 (15.38, 87.05)	<0.001
Light intensity (Fc/1000)	0.09 (0.02, 0.35)	0.001	0.14 (0.03, 0.58)	0.007
Grass present	5.81 (2.25, 14.99)	<0.001	2.79 (0.93, 8.34)	0.067
Fish present	3.80 (1.60, 9.01)	0.002	2.69 (0.92, 7.91)	0.071
Quarter (ref=July-September 2016)				
January-March 2016	3.04 (1.23, 7.51)	0.016	1.52 (0.49, 4.71)	0.465
April-June 2016	1.68 (0.69, 4.12)	0.254	1.08 (0.36, 3.24)	0.897
October-December 2016	2.89 (1.21, 6.91)	0.017	2.53 (0.89, 7.23)	0.083
January-March 2017	2.76 (1.11, 6.90)	0.030	1.34 (0.43, 4.17)	0.611
Organic bed material (ref=mixed, mud, or sand)	6.47 (1.62, 25.85)	0.008	3.49 (0.94, 12.92)	0.061
Amphibians present	2.40 (1.26, 4.56)	0.008	1.98 (0.90, 4.32)	0.088
Cloudy sky (ref=clear)	2.37 (1.23, 4.58)	0.010	-	-
Village: LIB, LUP, NHO, SAL or VIB (ref=SEM, TRI, or URC)	3.79 (1.27, 11.26)	0.017	-	-
Shade level (ref=none)				
Partial shade	2.63 (1.07, 6.43)	0.035	-	-
Total shade	2.60 (0.94, 7.23)	0.067	-	-
EVI (500m radius)	0.05 (0.003, 0.72)	0.028	0.01 (0.0003, 0.40)	0.014
Active fish pond (ref=abandoned fish pond or natural water body)	3.24 (1.16, 9.04)	0.025	-	-
Emergent vegetation present	1.88 (0.92, 3.83)	0.084	2.54 (1.06, 6.07)	0.036
Temperature (˚C)	0.81 (0.63, 1.04)	0.098	-	-
Number of people living in a 100m radius	1.02 (0.997, 1.04)	0.092	-	-
Percent forest loss 2010-2016 (500m radius)	1.27 (0.95, 1.68)	0.102	1.38 (1.06, 1.80)	0.016
Temporal water body (ref=permanent)	0.46 (0.16, 1.28)	0.137	-	-
Floating vegetation present	1.97 (0.71, 5.49)	0.195	-	-
Bushes present	1.62 (0.80, 3.28)	0.179	-	-
**Random effects**				
			ICC: 0.27	
			MOR: 2.86	

*Variables not associated with the presence of *Ny. darlingi* (bivariate logistic mixed-effects regression $p > 0.2$): alkalinity; conductivity; depth; distance to nearest forest; distance to *Ny. darlingi*-positive water body; distance to non-dry water body; hardness; NDWI (any buffer); pH; salinity; percent forest cover cover (any buffer); presence of algae or trees; water moving or turbid

Table IV: Results of bivariate and multivariate logistic mixed-effects models for the presence of *Ny. rangeli* larvae

Variable*	Bivariate Odds Ratio (95% CI)	Bivariate *p*-value	Multivariate Odds Ratio (95% CI)	Multivariate *p*-value
**Fixed effects**				
Intercept	-	-	0.003 (5x10^{-5}, 0.16)	0.004
Non-*Ny. rangeli* Anophelinae species present	100.54 (16.82, 600.97)	<0.001	138.08 (22.32, 854.31)	<0.001
Quarter (ref=July-September 2016)				
January-March 2016	1.17 (0.35, 3.90)	0.795	1.14 (0.27, 4.72)	0.862
April-June 2016	1.57 (0.49, 5.09)	0.449	2.12 (0.52, 8.59)	0.293
October-December 2016	1.83 (0.59, 5.62)	0.295	1.13 (0.28, 4.56)	0.864
January-March 2017	7.59 (2.44, 23.64)	<0.001	8.17 (1.98, 33.70)	0.004
Village: SAL or URC (ref=LIB, LUP, NHO, SEM, TRI, or VIB)	9.07 (3.29, 24.99)	<0.001	6.37 (1.98, 20.50)	0.002
Active fish pond (ref=abandoned fish pond or natural water body)	3.84 (1.43, 10.35)	0.008	-	-
Cloudy sky (ref=clear)	2.81 (1.26, 6.27)	0.011	-	-
Depth (m)	0.71 (0.55, 0.93)	0.013	-	-
Mixed bed material (ref=organic material, mud, or sand)	0.29 (0.11, 0.78)	0.014	-	-
Grass present	3.78 (1.17, 12.21)	0.026	-	-
Distance to nearest forest (m)	1.01 (1.003, 1.02)	0.014	1.01 (1.002, 1.02)	0.017
Bushes present	2.62 (1.15, 5.96)	0.022	2.92 (1.20, 7.10)	0.018
Amphibians present	2.19 (1.07, 4.52)	0.033	1.73 (0.78, 3.85)	0.176
Number of people living in a 100m radius	1.02 (1.00098, 1.04)	0.038	1.02 (1.004, 1.03)	0.013
EVI (500m radius)	0.04 (0.002, 0.80)	0.035	0.02 (0.0003, 1.26)	0.065
Fish present	2.95 (0.99, 8.75)	0.051	-	-
Trees present	2.00 (0.87, 4.58)	0.101	-	-
Percent forest cover (250m radius)	0.97 (0.94, 1.00)	0.090	-	-
Salinity (ppm)	1.03 (0.999, 1.07)	0.060	-	-
Water moving	0.37 (0.10, 1.32)	0.126	0.29 (0.08, 1.03)	0.056
Algae present	2.67 (0.77, 9.24)	0.121	3.78 (0.84, 17.01)	0.082
Light intensity (Fc/1000)	0.36 (0.09, 1.45)	0.150	-	-
Conductivity (uS/cm)	0.98 (0.96, 1.01)	0.186	-	-
Shade level (ref=none)				
Partial shade	2.27 (0.76, 6.74)	0.141	-	-
Total shade	1.63 (0.47, 5.61)	0.441	-	-
pH	1.38 (0.87, 2.18)	0.166	-	-
NDWI (250m radius)	0.02 (0.0001, 6.02)	0.187	-	-
**Random effects**				
			ICC: 0.05	
			MOR: 1.38	

*Variables not associated with the presence of *Ny. rangeli* (bivariate logistic mixed-effects regression $p > 0.2$): alkalinity; distance to *Ny. darlingi*-positive water body; distance to non-dry water body; hardness; percent forest loss 2010-2016 (any buffer); temperature; presence of floating or emergent vegetation; water body temporality; water turbid

Table V: Results of bivariate and multivariate logistic mixed-effects models for the presence of *Ny. triannulatus* s.l. larvae

Variable*	Bivariate Odds Ratio (95% CI)	Bivariate *p*-value	Multivariate Odds Ratio (95% CI)	Multivariate *p*-value
**Fixed effects**				
Intercept	-	-	0.0005 ($3x10^{-5}$, 0.007)	<0.001
Non-*Ny. triannulatus* s.l. Anophelinae species present	21.02 (7.57, 58.37)	<0.001	87.07 (18.38, 412.43)	<0.001
Quarter (ref=January-March 2016)				
April-June 2016	8.64 (1.47, 50.77)	0.017	9.65 (1.32, 70.37)	0.025
July-September 2016	11.70 (1.98, 69.05)	0.007	18.54 (2.34, 146.72)	0.006
October-December 2016	11.41 (2.00, 64.92)	0.006	8.72 (1.18, 64.38)	0.034
January-March 2017	79.24 (13.04, 481.47)	<0.001	261.11 (26.90, 2534.67)	<0.001
Village: SAL or URC (ref=LIB, LUP, NHO, SEM, TRI, or VIB)	8.13 (3.36, 19.68)	<0.001	16.45 (3.85, 70.23)	<0.001
Fish present	7.47 (1.86, 30.00)	0.005	-	-
Depth (m)	0.69 (0.53, 0.89)	0.004	-	-
Emergent vegetation present	0.34 (0.15, 0.74)	0.006	0.15 (0.04, 0.55)	0.004
Active fish pond (ref=abandoned fish pond or natural water body)	3.45 (1.50, 7.94)	0.004	-	-
Mud bed material (ref=organic material, sand, or mixed)	2.85 (1.28, 6.36)	0.011	-	-
Amphibians present	2.45 (1.23, 4.89)	0.011	2.53 (0.92, 6.95)	0.072
Floating vegetation present	3.68 (1.34, 10.10)	0.011	-	-
Distance to nearest forest (m)	1.01 (1.002, 1.02)	0.021	-	-
Bushes present	2.37 (1.09, 5.16)	0.030	-	-
pH	1.55 (1.004, 2.38)	0.048	-	-
Grass present	2.40 (0.86, 6.73)	0.096	-	-
Shade level (ref=none)				
Partial shade	1.92 (0.72, 5.13)	0.195	-	-
Total shade	1.07 (0.34, 3.39)	0.912	-	-
Water moving	0.39 (0.12, 1.31)	0.128	0.07 (0.01, 0.37)	0.002
Percent forest cover (500m radius)	0.97 (0.94, 1.01)	0.124	-	-
Salinity (ppm)	1.02 (0.99, 1.06)	0.183	-	-
Non-zero alkalinity (ref=0)	2.29 (0.80, 6.52)	0.121	-	-
EVI (500m radius)	0.11 (0.006, 1.91)	0.129	-	-
Any people living in a 50m radius	1.88 (0.72, 4.89)	0.199	-	-
**Random effects**				
			ICC: 0.33	
			MOR: 3.37	

*Variables not associated with the presence of *Ny. triannulatus* s.l. (bivariate logistic mixed-effects regression $p > 0.2$): cloud cover; conductivity; distance to *Ny. darlingi*-positive water body; distance to non-dry water body; hardness; percent forest loss 2010-2016 (any buffer); NDWI (any buffer); light intensity; presence of trees or algae; temperature; water body temporality; water turbid

Table VI: Results of bivariate and multivariate logistic mixed-effects models for the presence of *Ny.* sp. nr. *konderi* larvae

Variable*	Bivariate Odds Ratio (95% CI)	Bivariate *p*-value	Multivariate Odds Ratio (95% CI)	Multivariate *p*-value
**Fixed effects**				
Intercept	-	-	0.0002 ($6x10^{-6}$, 0.006)	<0.001
Non-*Ny.* sp. nr. *konderi* Anophelinae species present	18.79 (6.87, 51.36)	<0.001	14.09 (4.93, 40.25)	<0.001
Shade level (ref=none)				
Partial shade	7.95 (1.66, 38.13)	0.010	10.02 (1.54, 65.03)	0.016
Total shade	13.61 (2.67, 69.43)	0.002	12.57 (1.84, 85.89)	0.010
Light intensity (Fc/1000)	0.06 (0.009, 0.35)	0.002	0.23 (0.03, 1.65)	0.142
Quarter (ref=January-March 2017)				
January-March 2016	4.10 (1.32, 12.74)	0.015	4.52 (1.38, 14.80)	0.013
April-June 2016	3.85 (1.23, 12.04)	0.021	4.05 (1.22, 13.45)	0.023
July-September 2016	1.49 (0.43, 5.15)	0.527	2.31 (0.63, 8.50)	0.208
October-December 2016	2.08 (0.67, 6.42)	0.205	1.81 (0.57, 5.73)	0.316
Grass present	4.80 (1.45, 15.90)	0.010	-	-
Active fish pond (ref=abandoned fish pond or natural water body)	3.68 (1.45, 9.36)	0.006	2.40 (0.91, 6.31)	0.076
Organic bed material (ref=mixed, mud, or sand)	5.33 (1.50, 18.93)	0.010	-	-
Any people living in a 100m radius	0.35 (0.15, 0.85)	0.020	0.32 (0.13, 0.79)	0.014
Distance to nearest forest (m)	0.99 (0.97, 0.999)	0.033	0.99 (0.98, 1.00)	0.108
Cloudy sky (ref=clear)	2.51 (1.15, 5.47)	0.020	-	-
NDVI (100m radius)	10.02 (1.02, 97.97)	0.048	-	-
NDWI (100m radius)	102.07 (0.60, 17322)	0.077	-	-
Percent forest cover (250m radius)	1.03 (0.996, 1.06)	0.097	1.06 (1.02, 1.09)	0.001
Temperature (˚C)	0.79 (0.59, 1.06)	0.111	-	-
Percent forest loss 2010-2016 (500m radius)	1.22 (0.96, 1.54)	0.098	-	-
Bushes present	1.71 (0.77, 3.79)	0.184	-	-
Temporal water body (ref=permanent)	0.54 (0.21, 1.38)	0.198	-	-
**Random effects**				
			ICC: 0.01	
			MOR: 1.28	

*Variables not associated with the presence of *Ny.* sp. nr. *konderi* (bivariate logistic mixed-effects regression $p > 0.2$): village; alkalinity; conductivity; depth; distance to *Ny. darlingi*-positive water body; distance to non-dry water body; hardness; presence of fish or amphibians; pH; salinity; presence of algae, emergent vegetation, floating vegetation, or trees; water moving or turbid

Table VII: Number of reported cases of *Plasmodium vivax* and *Plasmodium falciparum* in each study village by season, 2016

Village	Population	2016 Annual Parasite Incidence (API) per 1000 inhabitants[1]	Rainy season, January-June		Dry season, July-December	
			Cases of *P. vivax*[1]	Cases of *P. falciparum*[1]	Cases of *P. vivax*[1]	Cases of *P. falciparum*[1]
LUP	381	659	101	71	34	45
NHO	228	171	35	1	2	1
TRI	245	78	14	1	4	0
SEM	207	130	24	1	2	0
LIB	264	367	59	27	7	4
SAL	318	311	70	22	1	6
URC	267	22	5	1	0	0
VIB	59	305	6	4	3	5
**Total**	1969		314	128	53	61

[1]Excludes repeat diagnoses within 60 days for *P. vivax*, 30 days for *P. falciparum*

Fig I: Median-joining *COI* haplotype network for members of the *Nyssorhynchus* Oswaldoi-Konderi complex from the current study and from GenBank (S2 Dataset). Circles represent unique haplotypes and are colored based on species (GenBank sequences) or sample origin (sequences from this study). The size of each circle is proportional to the number of individual sequences sharing the haplotype. Black nodes indicate theoretical missing haplotypes, and hash marks represent mutation steps between haplotypes.

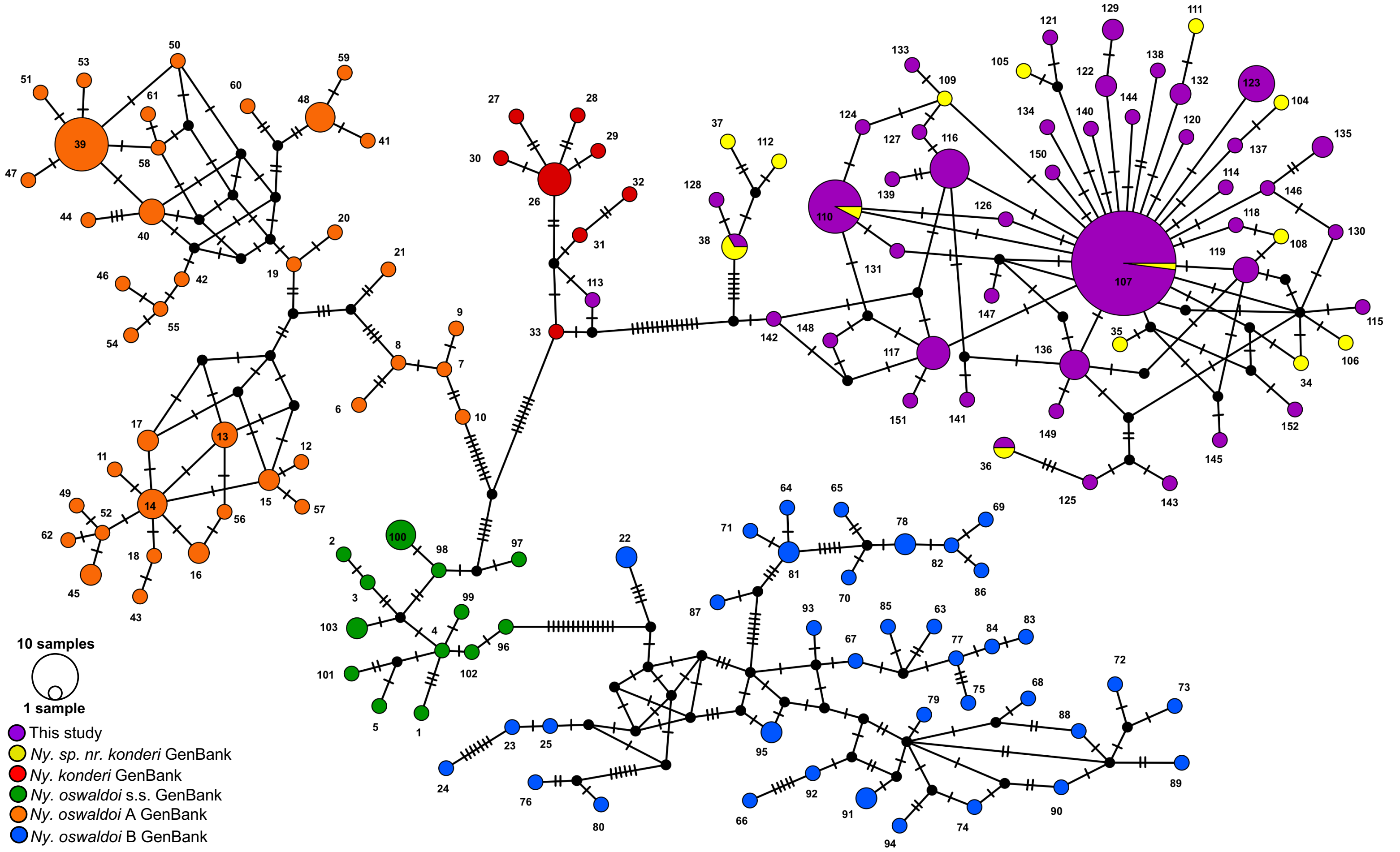

Fig II: Number and species of identified Anophelinae larvae by quarter in each village

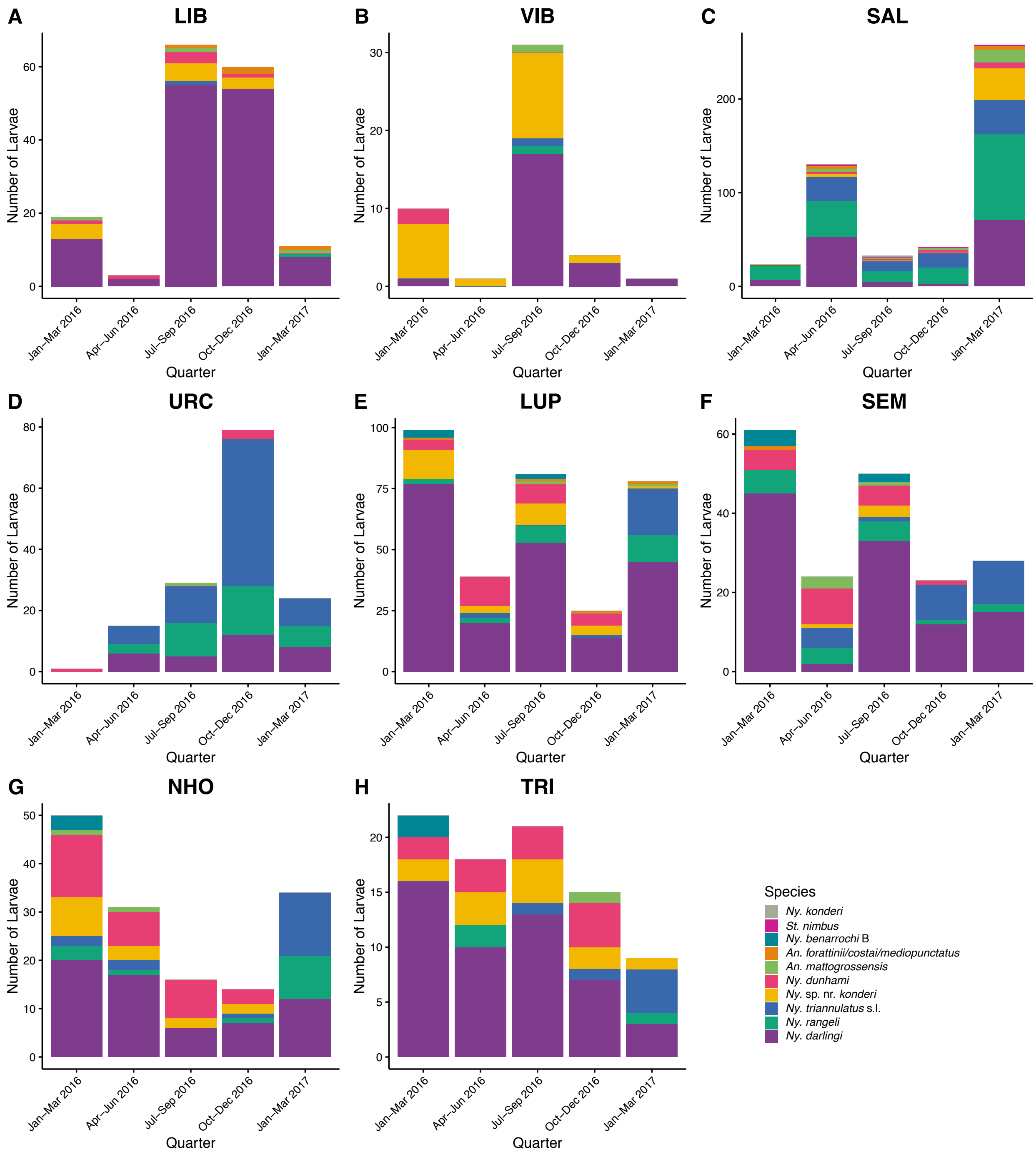

Fig III: Redundancy analysis (RDA) biplot showing the distribution of *Nyssorhynchus* species larvae in relation to environmental variables. The proportion of total variance in the species presence matrix explained by each axis is shown in brackets. The position of each mosquito species name (shown in red) and environmental variable name (shown in blue) indicates its biplot score; arrows are not shown for clarity.

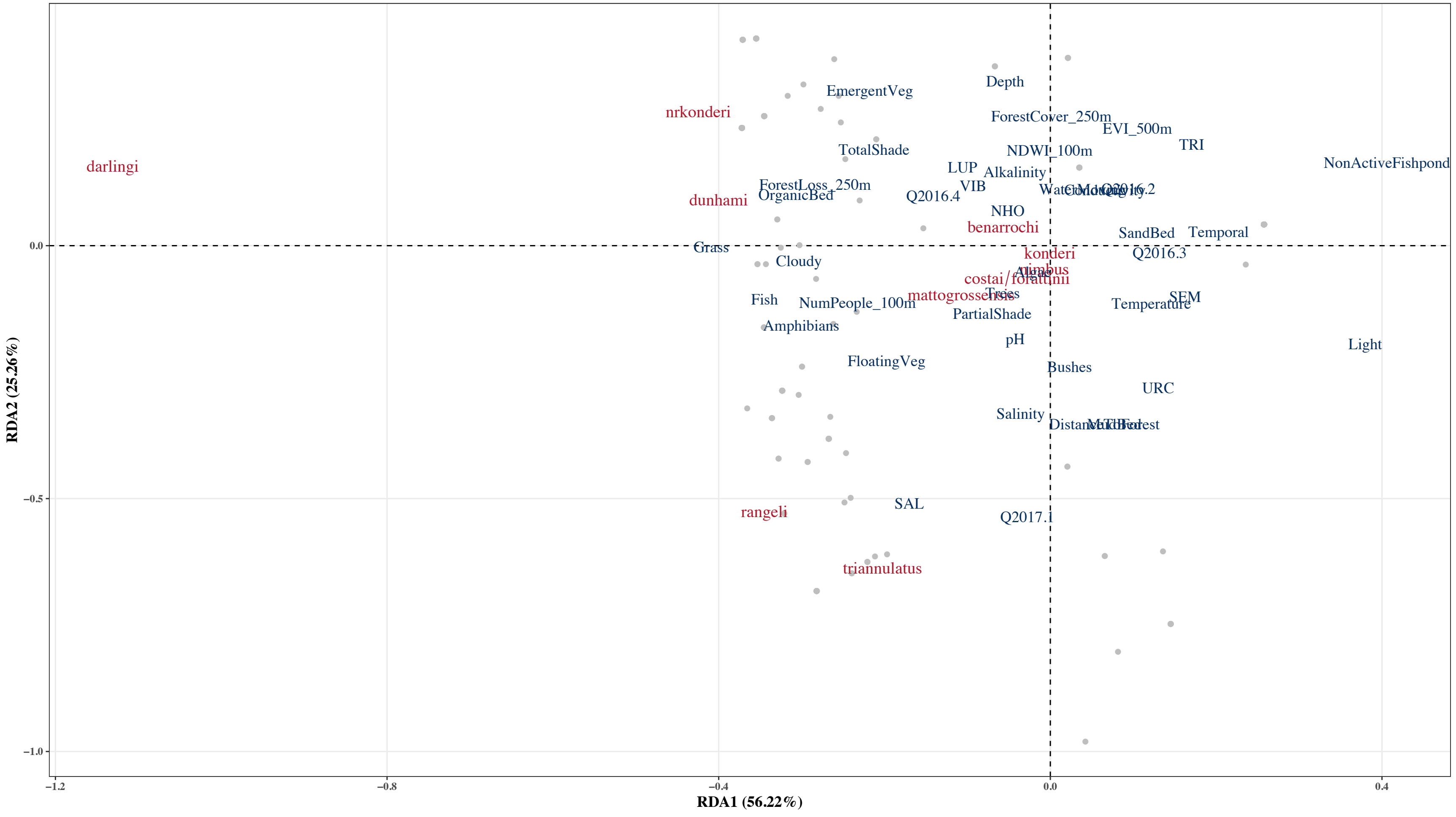

Fig IV: Alpha rarefaction curve from bacterial 16S rRNA sequencing, generated by sampling between 1 and 20,000 reads per larva, increasing in increments of 2,000 until the total number of reads per sample was reached. Ten iterations were computed at each step, and the smoothed mean number of OTUs observed for each larva at each step are shown.

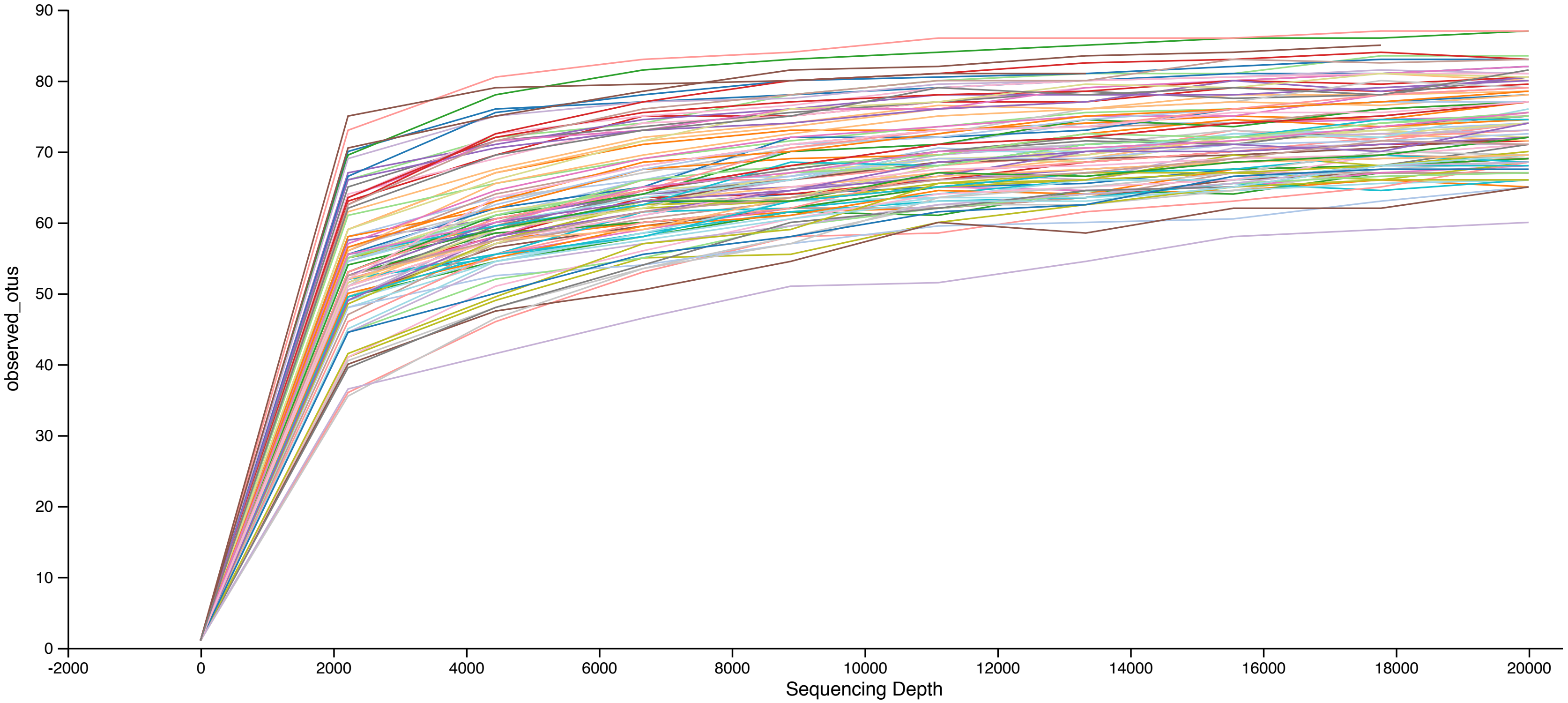

Fig V: Principal Coordinates Analysis (PCoA) of (A) weighted UniFrac, (B) Bray-Curtis distance matrices of *Nyssorhynchus* larval bacterial communities, with individual larvae colored by species. Ellipses indicate 95% confidence intervals around each species.

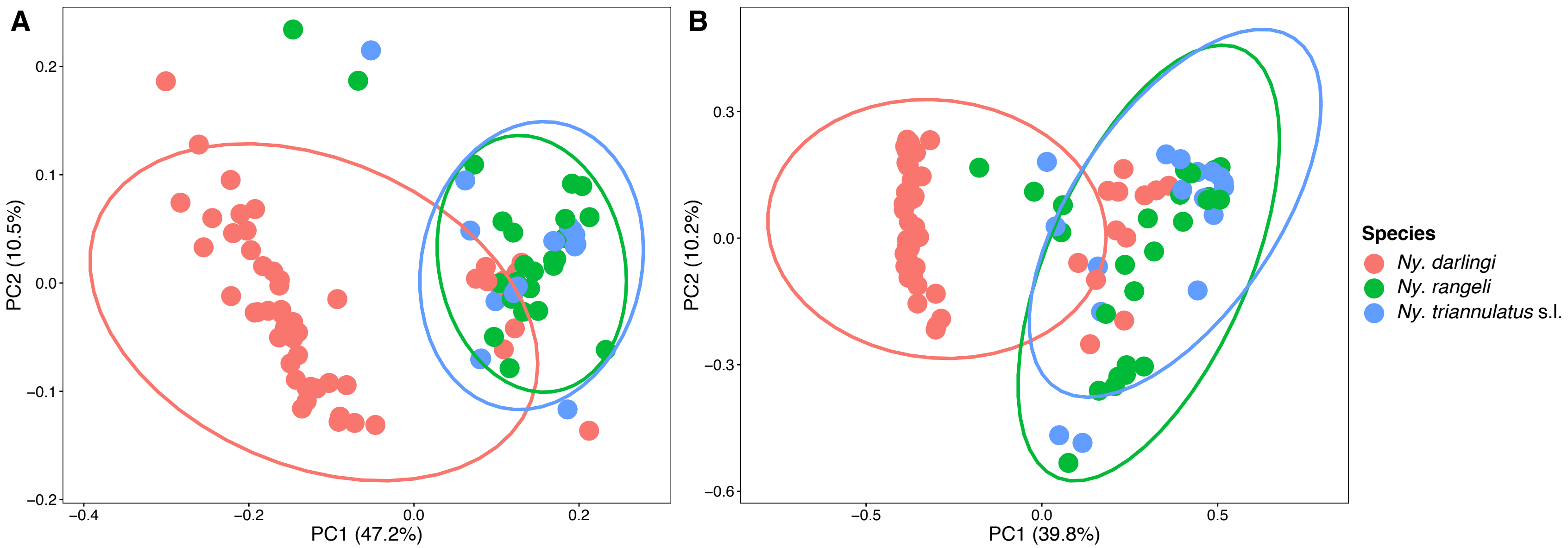

Fig VI: Principal Coordinates Analysis (PCoA) of (A) unweighted UniFrac, (B) weighted UniFrac, (C) Bray-Curtis distance matrices of *Nyssorhynchus* larval bacterial communities, with individual larvae colored by water body. Confidence intervals are not shown for clarity.

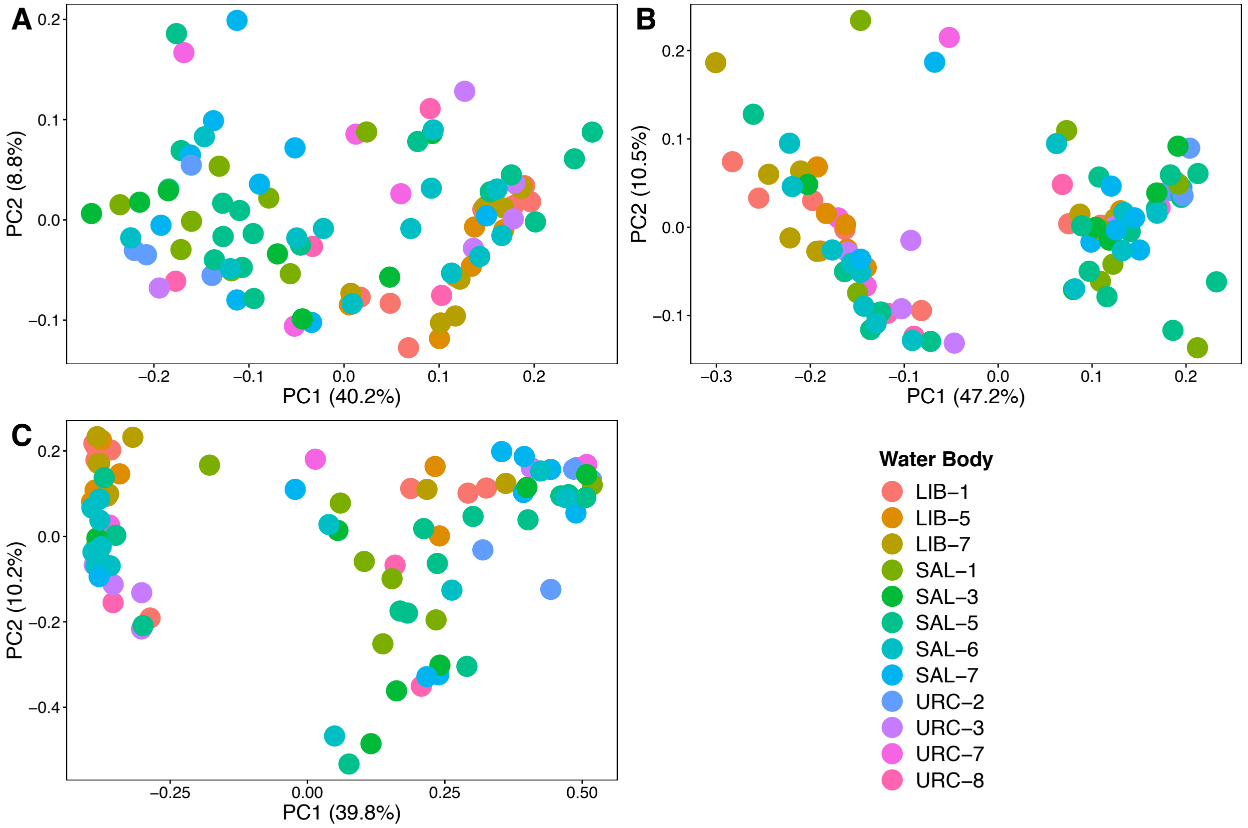

Fig VII: Bacterial family composition of each *Nyssorhynchus* larva, ordered by larval species (labels on top), then by water body ID (labels on bottom)

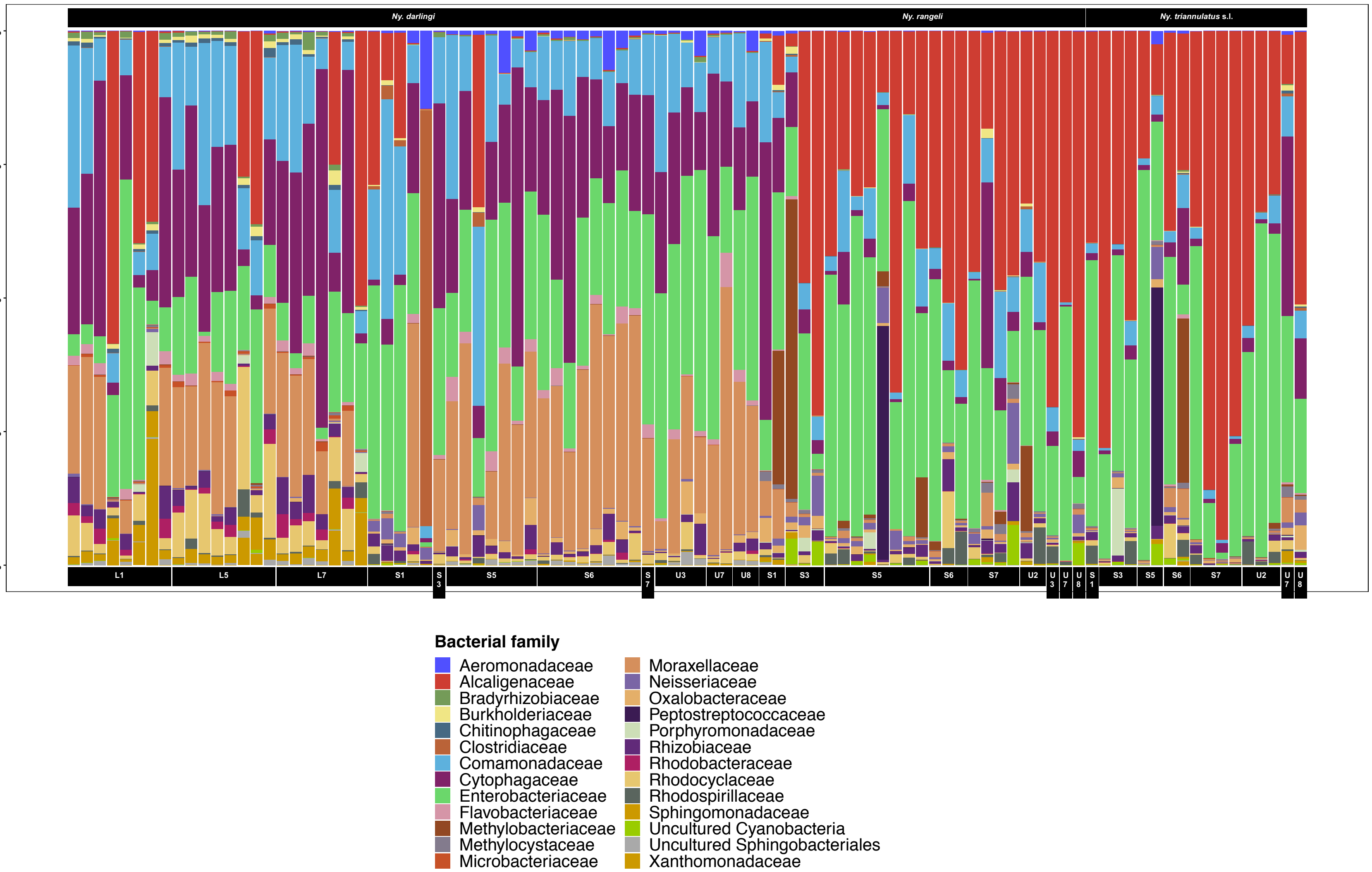

Supplement: S1 File — Contains Tables I-VII and Figures I-VII. Table I: Primer sequences. • Table II: Dates of Google Earth imagery used for distance to nearest forest calculations. • Table III: Results of bivariate and multivariate logistic mixed-effects models for the presence of Ny. darlingi larvae. • Table IV: Results of bivariate and multivariate logistic mixed-effects models for the presence of Ny. rangeli larvae. • Table V: Results of bivariate and multivariate logistic mixed-effects models for the presence of Ny. triannulatus s.l. larvae. • Table VI: Results of bivariate and multivariate logistic mixed-effects models for the presence of Ny. sp. nr. konderi larvae. • Table VII: Number of reported cases of Plasmodium vivax and Plasmodium falciparum in each study village by season, 2016. • Fig I: Median-joining COI haplotype network for members of the Nyssorhynchus Oswaldoi-Konderi complex. • Fig II: Number and species of identified Anophelinae larvae by quarter in each village. • Fig III: Redundancy analysis (RDA) biplot showing the distribution of Nyssorhynchus species larvae in relation to environmental variables. • Fig IV: Alpha rarefaction curve from bacterial 16S rRNA sequencing. • Fig V: Principal Coordinates Analysis (PCoA) of (A) weighted UniFrac, (B) Bray-Curtis distance matrices of Nyssorhynchus larval bacterial communities, with individual larvae colored by species. • Fig VI: Principal Coordinates Analysis (PCoA) of (A) unweighted UniFrac, (B) weighted UniFrac, (C) Bray-Curtis distance matrices of Nyssorhynchus larval bacterial communities, with individual larvae colored by water body. • Fig VII: Bacterial family composition of individual Nyssorhynchus larva. (PDF) [file pntd.0007412.s001.pdf]
